# Enteric innervation combined with proteomics for the evaluation of the effects of chronic fluoride exposure on the duodenum of rats

**DOI:** 10.1038/s41598-017-01090-y

**Published:** 2017-04-21

**Authors:** Carina Guimarães de Souza Melo, Juliana Vanessa Colombo Martins Perles, Jacqueline Nelisis Zanoni, Sara Raquel Garcia de Souza, Erika Xavier Santos, Aline de Lima Leite, Alessandro Domingues Heubel, Camila Oliveira e Souza, Juliana Gadelha de Souza, Marília Afonso Rabelo Buzalaf

**Affiliations:** 1 Department of Biological Sciences, Bauru School of Dentistry, University of São Paulo, Bauru, Brazil; 2 Department of Morphophysiological Sciences, State University of Maringá, Paraná, Brazil

## Abstract

Ingested fluoride (F) is absorbed mainly in the small intestine, which is controlled by the Enteric Nervous System (ENS). Although important intestinal symptomatology has been described after excessive F exposure, there have been no studies reporting the effects of F on the ENS. In this study, the effects of chronic F exposure were evaluated on the duodenums of rats through proteomic and morphological analyses. Concentrations of 0, 10, or 50 ppm of F were applied to the drinking water for 30 days. Immunofluorescence techniques were performed in the myenteric plexus of the duodenum to detect HuC/D, neuronal nitric oxide (nNOS), vasoactive intestinal peptide (VIP), calcitonin gene related peptide (CGRP), and substance P (SP). The 50 ppm F group presented a significant decrease in the density of nNOS-IR neurons. Significant morphological alterations were also observed in HUC/D-IR and nNOS-IR neurons; VIP-IR, CGRP-IR, and SP-IR varicosities for both groups (10 and 50 ppm F). Proteomic analysis of the duodenum demonstrated alterations in the expression of several proteins, especially those related to important biological processes, such as protein polymerization, which helps to explain the downregulation of many proteins upon exposure to 50 ppm of F.

## Introduction

The gastrointestinal tract (GIT) is considered the main route of fluoride (F) exposure, and it receives on a daily basis different F concentrations due to food and water ingestion^1^. The stomach is an important site of F absorption, and even with the low gastric pH of 1.5, at which F is quickly absorbed as undissociated molecule (HF)^2^, the stomach is responsible for no more that 40% of its absorption^3^. In the small intestine, the rate of absorption is significantly higher, and it is not pH-dependent as in the stomach^2, 4^, ranging from 45%^5^ to 75%^4^ of the total absorption.

Gastrointestinal symptoms have been described as important signs of F toxicity^6, 7^, and complaints such as nausea, vomiting, diarrhoea and abdominal pain have been reported^8^ in at least in 70% of patients evaluated after long-term ingestion of F^9^. Specially in the regions of endemic fluorosis, numbering at least 22 countries^10^, loss of appetite, flatulence, constipation, intermittent diarrhoea mimicking irritable bowel syndrome^7, 11^, and even colitis^12^ have also been described as consequences of fluorosis.

The function of the gastrointestinal tract (GIT) is controlled by an interconnected set of neurons in the walls of its organs, named the enteric nervous system (ENS)^13^. Alterations in the ENS can affect the patterns of motility, absorption, secretion and permeability^14^, compromising GIT behavior^15^. The ENS presents a reflex activity working independently of the central nervous system (CNS), which is affected by excessive F exposure^16^. The toxic effects of excessive concentrations of F on the CNS have been described and correlated with behavioural^17^ and cognitive deficits^18, 19^, neural dysfunctions^20^ and morphological alterations^21–23^.

Although the toxicity of F on the CNS has been described in great detail and the GIT is considered an important site of F absorption, there is no information regarding the effects of F on the ENS, even with relevant GIT symptomatology reported after excessive F exposure. Specifically on the myenteric plexus, which is responsible for the control of the intestinal motility, this pioneer study investigated how the morphology of different types of enteric neurons of the duodenum can be affected by chronic F exposure. Additionally, to understand the mechanisms involved in the effects of F on the intestine, proteomic analysis was employed to evaluate alterations in the protein expression of the duodenum wall since the proximal portion of the intestine is considered the main site of F absorption^4, 24^.

## Methods

### Materials

The materials used included hexamethyldisiloxane (HMDS, Sigma-Aldrich, St. Louis, MO, USA); sodium fluoride (NaF, Sigma Chemical, St. Louis, MO, USA); trypsin (Trypsin Gold Mass Spectrometry, Promega, Madison, USA); *PlusOne 2D Cleanup* kit (GE Healthcare, Uppsala, Sweden) and 3 kDa AMICON (Millipore, St Charles, MO, USA). Antibodies: nNOS (H-299, sc-8309 Santa Cruz, Dallas, TX, USA), Mouse anti-HuC/D (A-21271, Invitrogen, Waltham, MA, USA), Rabbit anti-CGRP (AB15360, Millipore, St Charles, MO, USA), Rabbit anti-VIP (V0390, Sigma-Aldrich, St. Louis, MO, USA), Goat anti-substance P (sc9758, Santa Cruz, Dallas, TX, USA), Anti-rabbit 546 (A10040, Invitrogen, Waltham, MA, USA), Anti-mouse 488 (A21202, Invitrogen, Waltham, MA, USA), Anti-rabbit 546 (A10040, Invitrogen, Waltham, MA, USA), and Anti-goat 568 (A11057, Invitrogen, Waltham, MAs, USA).

### Sample collection and plasma F concentrations analysis

Plasma F concentrations were determined after overnight hexamethyldisiloxane (HMDS)-facilitated diffusion^25^ as previously described^26^. Data were analysed by ANOVA (after log transform) and Tukey’s test using GraphPad InStat software, version 3.0 for Windows (GraphPad Software Inc., La Jolla, CA, USA). The significance level was set at 5%.

### Animals

The protocol of this study was previously approved by the Research Ethics Committee of Bauru Dental School, University of São Paulo (protocol 014/2011). All of the experiments were performed in accordance with guidelines of the National Research Council. The study was conducted on 18 male rats (*Rattus norvegicus*, Wistar type). The animals were housed individually in metabolic cages having access to water and food *ad libitum*. The lighting schedule in the housing room was 12 hours of light and 12 hours of darkness per day. The room temperature was controlled (22 ± 2 °C). The animals were randomly divided into 3 groups (n = 6 per group). They were chronically exposed to NaF in their drinking water for 30 days at concentrations of 0 ppm of F (deionized water), 10 ppm of F or 50 ppm of F. Due to the fast metabolism of F in rodents, the concentration of 10 ppm of F corresponds to 1–2 ppm added to the public water supplies used for drinking water^27^, which allowed us to compare the results obtained with values ingested by humans on a daily basis. After the experimental period of exposure, the animals had their blood harvested for quantification of F and the duodenum harvested for histological, immunofluorescence and proteomic analyses.

### Histological analysis

The intestinal lumen was washed, fixed in 10% neutral-buffered formalin, embedded in paraffin, cut into 5 µm sections and stained with haematoxylin and eosin (HE) using a standard technique. Images were obtained (10X objective) using a high resolution camera (Moticam 2500, Motic China Group Co, Shanghai, China) coupled to a microscope (Olympus BX40, Olympus Co., Japan). The thickness of the tunica muscularis and of the total wall of the duodenum were measured using Image-Pro Plus software in 10 sections per animal in 5 different regions (images not provided).

### Myenteric plexus immunofluorescence

After laparotomy the duodenum was washed with 0.1 M PBS (pH 7.4) and filled with Zamboni’s fixative^28^ for 18 h at 4 °C. This segment was opened along the mesenteric border and washed in 80% ethanol to remove the fixative. The samples of duodenum were then dehydrated in an increasing ethanol series (95% and 100%), cleared with xylene, gradually rehydrated in a decreasing ethanol series (100%, 90%, 80%, 50%), and stored in PBS containing 0.08% sodium azide at 4 °C. The samples were cut into pieces measuring approximately 1 cm^2^ and were microdissected under a Stemi DV4 stereo microscope (Zeiss, Jena, Germany) to obtain whole-mount preparations of the tunica muscularis (since study of the myenteric plexus requires removal of the mucosal and submucosal layers). The whole mounts were processed for immunofluorescence techniques to detect Human neuronal protein (HuC/D), Vasoactive Intestinal Peptide (VIP), Calcitonin gene*-*related peptide (CGRP), and Substance P (SP). The HuC/D was used, since it is an important marker of the general enteric neuronal population^29^. To identify the neurons that present Nitric Oxide (NO) as a neurotransmitter, an antibody for the neuronal Nitric Oxide (nNOS) was applied^15^, and the immunoreactive cell bodies were named HuC/D-IR and nNOS-IR, respectively. In the myenteric plexus cell bodies of neurons that present VIP are rarely found, but present varicose axons that also exhibits immunoreactivity to VIP. So, in the present study, the VIP neurons were identified by their varicosities (VIP-IR), as also CGRP and SP neurons (CGRP-IR and SP-IR, respectively). For the immunofluorescence techniques, the samples were processed as described by Hermes-Uliana *et al*.^30^. Briefly, the dissected samples were washed in PBS with 0.5% Triton X-100 and were incubated for 1 h in blocking solution (PBS with 0.5% Triton X-100, 2% BSA, and 10% donkey serum). For the double-labelling technique, the following primary antibodies, in determined concentrations, were applied for 48 hours: anti-HuC/D (mouse, 1:500), and anti-nNOS (rabbit, 1:500). After this first incubation period, the samples were washed 3 times in PBS and incubated in secondary antibodies [Alexa Fluor 488 (1:250), Alexa Fluor 568 (1:500)]. After incubation, the samples were washed 3 times in PBS and mounted on slides using Prolong Gold antifade. For each animal, 4 slides were prepared: one for the HuC-D/nNOS technique and one for the other 3 techniques separately (VIP, CGRP, and SP).

### Image capture

The images for morphometric and quantitative analysis were obtained using an AxioCam digital camera (Zeiss, Jena, Germany) coupled to an Axioskop Plus light microscope (Zeiss). AxioVision software, version 4.1, a Zeiss specific software, was used to scan the images. For each technique (HuC-D/nNOS, VIP SP, and CGRP), 32 images were captured/animal. Image-Pro Plus software, version 4.5.0.29 (Media Cybernetics, Silver Spring, MD, USA), was used to analyse the images. Additionally, high resolution images were obtained with the spectral confocal microscope Leica TCS SPE (Leica Microsystems CMS, Heerbrugg-Switzerland). Both microscopes are property of the Integrated Research Centre (CIP/FOB/USP/Bauru, Brazil).

### Morphometric and quantitative analysis

The quantitative analysis was performed for the HuC-D/nNOS technique based on the neuronal density (neurons/cm^2^) and the morphometric analysis based on the area (µm^2^) of 100 neuronal cell bodies/animal. For the VIP, SP and CGRP techniques the morphometric analysis was performed in 400 varicosities from each animal (2,400/group). Images were obtained using a 20X microscope objective for the HuC-D/nNOS technique and a 40X objective for the VIP, SP and CGRP techniques. All images were analysed using the Image-Pro Plus ® software, version 4.5.0.29 (Media Cybernetics, Silver Spring, MD, USA). The results were statistically evaluated using Statistica software, version 7.1® (*Statsoft Inc*., 2005, Tulsa, OK, USA), and GraphPad Prism software, version 3.1 (GraphPad Software, San Diego, CA). Delineation blocks and Tukey’s test were necessary to evaluate the morphometric data. For the quantitative values, ANOVA and Tukey’s test were applied. Statistically significant values are presented as p < 0.05.

### Sample preparation for proteomic analysis

The duodenum was harvested and frozen in liquid nitrogen. The frozen samples were homogenized in a cryogenic mill (model 6770, Spex, Metuchen, NJ, USA). Protein preparation for proteomic analysis was performed as previously described^31^. Briefly, proteins were extracted by incubation in lysis buffer (7 M urea, 2 M thiourea, 4% CHAPS, 1% IPG buffer pH 3–10, 40 mM DTT) for 1 h at 4 °C and then were precipitated using the *PlusOne 2D Cleanup* kit. Duodenum proteins (25 µL) from each animal of the same group were combined to constitute a pool. Then 50 mM AMBIC containing 3 M urea was added to each pool, the sample was filtered in 3 kDa AMICON and the protein was quantified using the Bradford method^31^. Samples were reduced (DTT), alkylated (IAA) and digested with 100 ng trypsin for 14 h at 37 °C. After digestion 10 µl of 5% TFA were added, the sample was incubated for 90 min at 37 °C and centrifuged (14,000 rpm for 30 min). The supernatant was harvested, and 5 µL of ADH (1pmol/µL) plus 85 µL of 3% ACN were added.

### LC-MS/MS and bioinformatics analyses

The peptide identification was performed on a nanoAcquity UPLC-Xevo QTof MS system (Waters Corporation, Manchester, UK), as previously described^32^. Differences in expression among groups were obtained using the ProteinLynx Global Server (PLGS) software provided by Waters Corporation and are expressed as p < 0.05 for downregulated proteins and 1 − p > 0.95 for upregulated proteins. Bioinformatics analysis was performed for comparison of the groups exposed to F relative to the control group (Tables S1–S6), as reported earlier^32–35^.

## Results

### Plasma F concentrations

The validation of the exposure was performed through analysis of the plasma F concentrations. Significant differences were found among the groups (F = 53.57, p < 0.0001; Table 1), confirming the exposure. Both groups exposed to F (10 and 50 ppm F) presented plasma F concentrations significantly higher than in the control group.

**Table 1:** Average Plasma fluoride concentration (μg/mL ± SD) in rats exposed or not to F chronically, through the drinking water for 30 days.

GROUPS	[F] plasma (μg/mL ± SD)
Control	0.011 ± 0.003^a^
10 ppm F	0.021 ± 0.010^b^
50 ppm F	0.036 ± 0.012^c^

Animal groups: Control (deionized water–0 ppm F), 10 ppm F, and 50 ppm de F. *Different letter indicated significant difference among the groups (ANOVA after log transformation and Tukey’s test (p < 0.001)). n = 6.

### Morphological analysis of the duodenum wall thickness

The mean (±SD) thickness of the duodenum *tunica muscularis* was significantly increased in the 50 ppm F group (161.55 ± 3.73 µm^2^) compared to control (129.65 ± 1.73 µm^2^) and the 10 ppm F group (137.17 ± 2.24 µm^2^) (p < 0.05). However, the total thickness of the duodenum wall did not significantly differ among the groups (data not shown).

### Morphometric and quantitative analysis of myenteric neurons of the duodenum

#### Myenteric neurons HuC/D-IR analysis

In the morphometric analysis of the general population of neurons, the cell body areas (µm^2^) of the HuC/D-IR neurons of the duodenum presented a statistically significant difference among the groups (p < 0.05), with a decrease in the average value of the areas for the 50 ppm F group, compared to the control group (p < 0.05). In the quantitative analyses, no significant change was observed in the density of the HuC/D-IR neurons (p > 0.05, Table 2).

**Table 2:** Means and standard errors of the values of the cell bodies areas and density of the HUC/D-IR myenteric neurons of the duodenum of rats exposed or not to F chronically, through the drinking water for 30 days.

GROUPS	Cell bodies areas of the HuC/D-IR neurons (µm^2^)	Density HuC/D-IR neurons (neurons/cm^2^)
Control	288.6 ± 3.2^a^	13,663.3 ± 290.9^a^
10 ppm F	286.0 ± 3.6^a^	13,823.6 ± 390.8^a^
50 ppm F	263.2 ± 3.1^b^	13,872.8 ± 319.2^a^

Animal groups: Control (deionized water-0 ppm F), 10 ppm F, and 50 ppm de F. In the quantitative analysis the Fisher’s test was applied but no significant difference was observed among the groups. In the morphometric analysis, means followed by different letters in the same column are statistically different according to Tukey’s test (p < 0.05).

#### Myenteric nNOS-IR neuron analysis

In the morphometric analyses of the nNOS-IR neurons the values of the cell body areas showed the same pattern as the HuC/D–IR neurons, with a decrease in the mean value of the areas for the 50 ppm F group compared to the control group (p < 0.05). In the quantitative analyses, a significant reduction in the density of nNOS-IR neurons was found for the 50 ppm F group, compared to the control group (p < 0.05) (Table 3).

**Table 3:** Means and standard errors of the values of the cell bodies areas and density of the nNOS-IR myenteric neurons of the duodenum of rats exposed or not to F chronically, through the drinking water for 30 days.

GROUPS	Cell bodies areas of the nNOS-IR neurons (µm^2^)	Density nNOS-IR neurons (neurons/cm^2^)
Control	281.8 ± 2.90^a^	5,339.6 ± 125.2^a^
10 ppm F	274.9 ± 2.97^ab^	5,257.1 ± 135.0^a^
50 pm F	267.5 ± 2.99^b^	4,706.3 ± 124.4^b^

Animal groups: Control (deionized water–0 ppm F), 10 ppm F, and 50 ppm de F. In the quantitative analysis the Fisher’s test was applied but no significant difference was observed among the groups. In the morphometric analysis, means followed by different letters in the same column are statistically different according to Tukey’s test (p < 0.05).

#### Myenteric varicosities VIP-IR, CGRP-IR, or SP-IR morphometric analysis

In the morphometric analyses of VIP-IR varicosity area (µm^2^), a significant increase was detected in both groups exposed to F (10 and 50 ppm F), compared to the control group (p < 0.05 when all groups were compared) (Table 4). In the morphometric analyses of the CGRP-IR varicosity areaa (µm^2^), a significant increase was observed in the 10 ppm F group and a decrease was observed in the 50 ppm F group, compared to the control group (p < 0.05) (Table 4). In the morphometric analyses of SP-IR varicosity areas (µm^2^), a significant increase was observed in both groups exposed to F, compared to the control group (p < 0.05) (Table 4). Representative images of the immunofluorescence are displayed in the supplementary information (Supplementary Figs S1 and S2).

**Table 4:** Means and standard errors of the VIP-IR, CGRP-IR, and SP-IR values of myenteric neurons varicosities areas of the duodenum of rats exposed or not to F chronically, through the drinking water for 30 days.

GROUPS	Area VIP-IR varicosities (µm^2^)	Area CGRP-IR varicosities (µm^2^)	Area SP-IR varicosities (µm^2^)
Control	2.64 ± 0.02^a^	3.44 ± 0.03^a^	3.18 ± 0.03^a^
10 ppm F	2.94 ± 0.03^b^	3.58 ± 0.03^b^	4.34 ± 0.02^b^
50 ppm F	2.93 ± 0.03^b^	3.25 ± 0.03^c^	5.16 ± 0.03^c^

Animal groups: Control (deionized water–0 ppm F), 10 ppm F, and 50 ppm de F. Means followed by different letters in the same column are statistically different according to Tukey’s test (p < 0.05). n = 6.

#### Proteomic analysis of the duodenum

**Figure 1:**
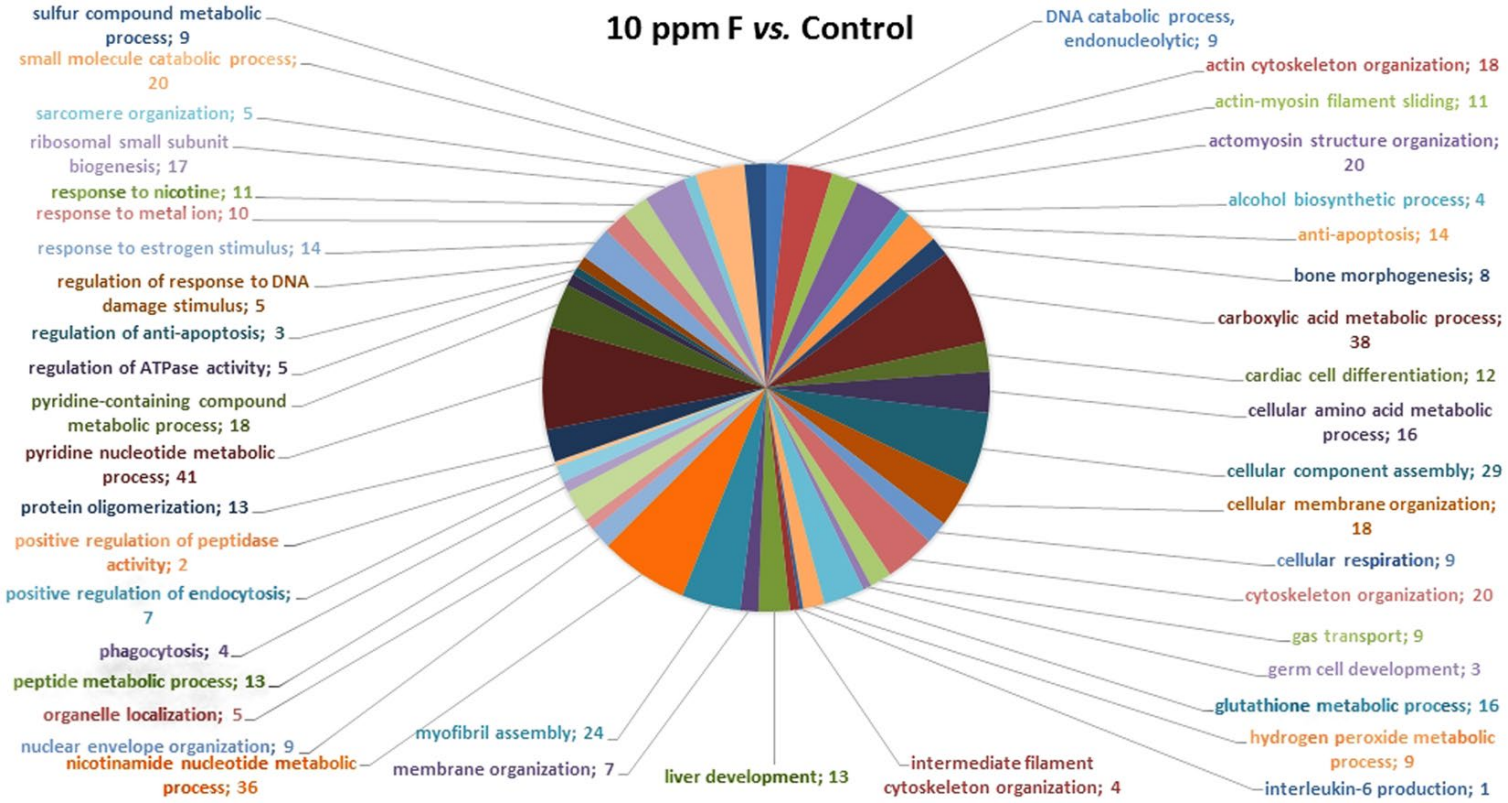
Functional distribution of proteins identified with differential expression in the duodenum of rats exposed chronically to 10 ppm F vs. Control Group (0 ppm F). Categories of proteins based on GO annotation Biological Process. Terms significant (Kappa = 0.04) and distribution according to percentage of number of genes association. Proteins access number was provided by the UNIPROT. The gene ontology was evaluated according to ClueGo® pluggins v2.0.7 of Cytoscape® software^36, 37^.

**Figure 2:**
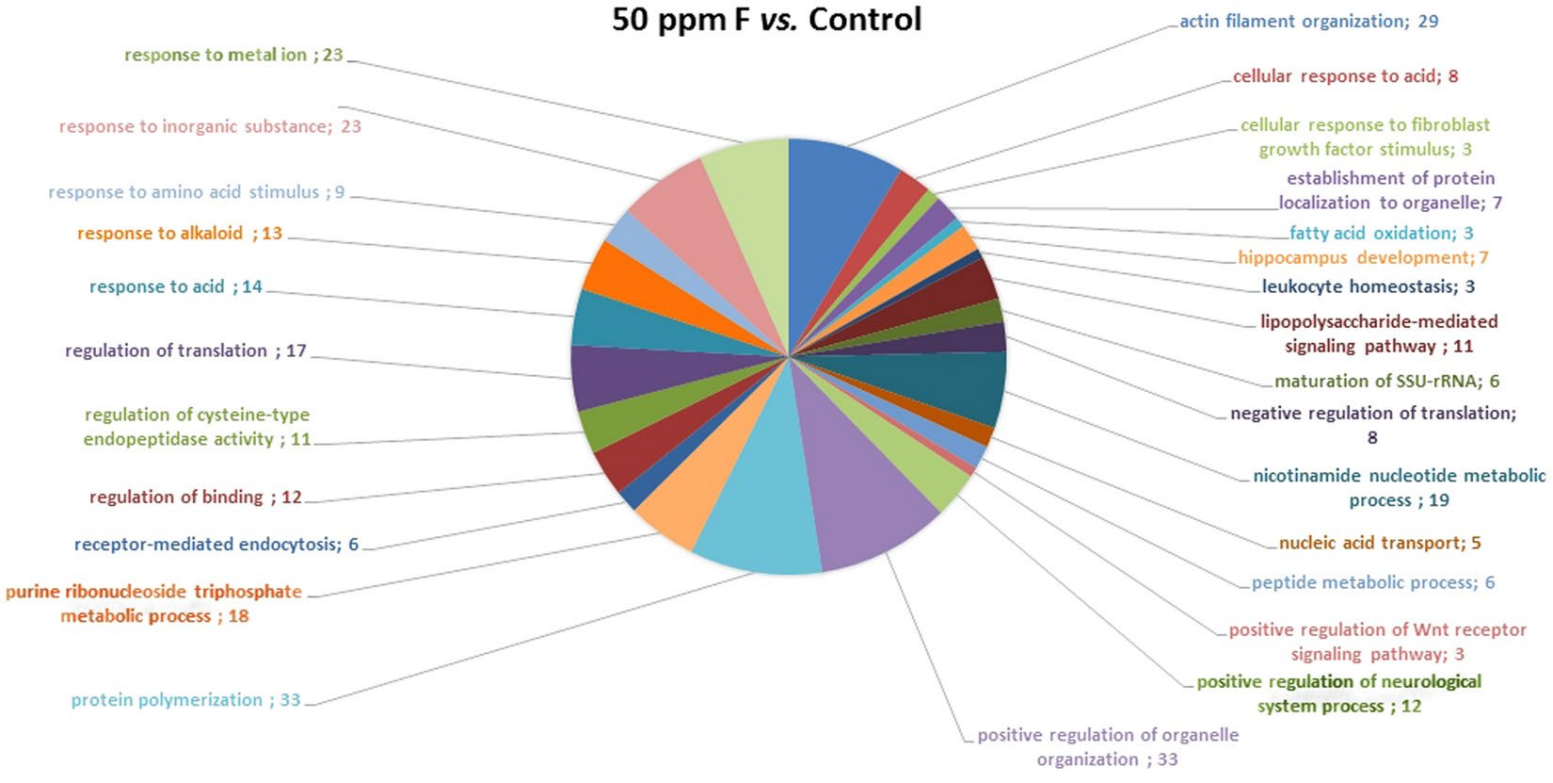
Functional distribution of proteins identified with differential expression in the duodenum of rats exposed chronically to 50 ppm F vs. Control Group (0 ppm F). Categories of proteins based on GO annotation Biological Process. Terms significant (Kappa = 0.04) and distribution according to percentage of number of genes association. Proteins access number was provided by the UNIPROT. The gene ontology was evaluated according to ClueGo® pluggins v2.0.7 of Cytoscape® software^36, 37^.

**Figure 3:**
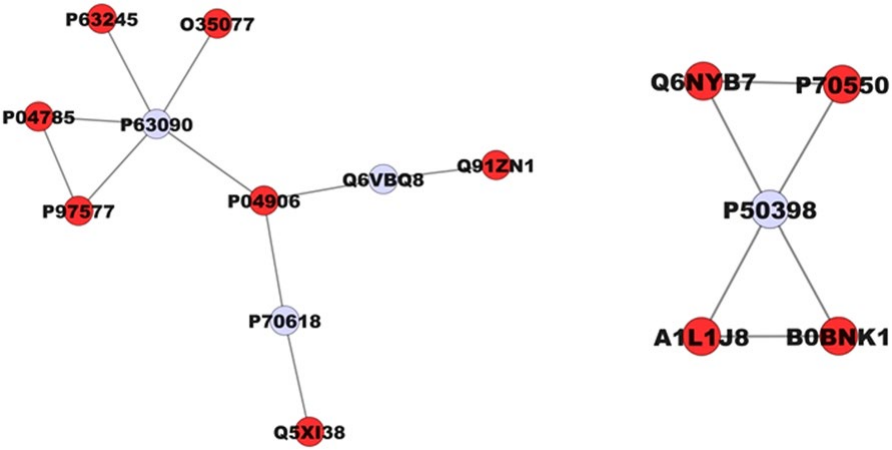
Subnetworks generated by VizMapper for each comparison −10 ppm F vs. Control Group. Color of node indicates the differential expression of the respective protein, for each comparison. Red nodes indicate protein down-regulation. Gray node indicates proteins presenting interaction but that were not identified in the present study. The access numbers in grey nodes correspond to: Pleiotrophin (P63090), RNA-binding protein PNO1 (Q6VBQ8), Mitogen-activated protein kinase 14 (P70618), and Rab GDP dissociation inhibitor alpha (P50398). The access numbers in red nodes correspond to: *Guanine nucleotide-binding protein subunit beta-2-like 1* (P63245), *Glycerol-3-phosphate dehydrogenase* (O35077), *Protein disulfide-isomerase* (P04785), *Fasciculation and elongation protein zeta-1* (P97577), *Glutathione S-transferase P* (P04906), *Coronin-1A* (Q91ZN1), *Lymphocyte cytosolic protein 1* (Q5XI38), *Ras-related protein Rab-1A* (Q6NYB7), *Ras-related protein Rab-8B* (P70550), *Protein Rab5b* (A1L1J8), and *Protein Rab5c* (B0BNK1).

**Figure 4:**
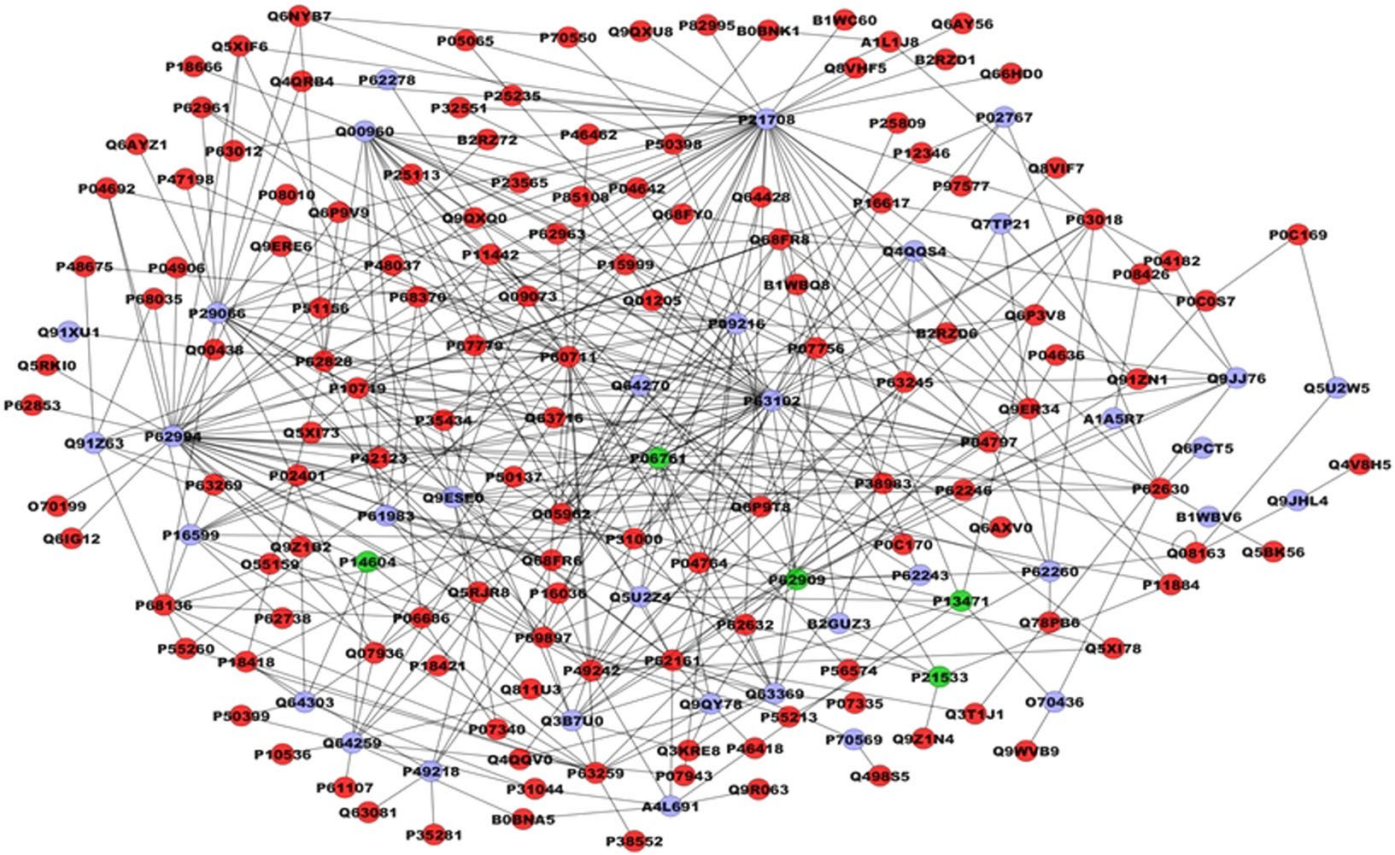
Subnetworks generated by VizMapper for each comparison −50 ppm F vs. Control Group. Color of node indicates the differential expression of the respective protein, for each comparison. Red nodes indicate protein down-regulation. Gray node indicates proteins presenting interaction but that were not identified in the present study. The access numbers in grey nodes correspond to: Putative RNA exonuclease NEF-sp (A1A5R7), PIN2/TERF1-interacting telomerase inhibitor 1 (A4L691), Aplp1 protein (B1WBV6), Mthfd1l protein (B2GUZ3), Mothers against decapentaplegic homolog 2 (O70436), Transthyretin (P02767), Protein kinase C epsilon type (P09216), Tumor necrosis fator-TNF (P16599), Mitogen-activated protein kinase 3-Mapk3 (P21708), Beta-arrestin-1 (P29066), Melatonin receptor type 1A (P49218), 14-3-3 protein gamma (P63102), 40S ribosomal protein S8 (P62243), 14-3-3 protein epsilon (P62260), 40S ribosomal protein S13 (P62278), Growth factor receptor-bound protein 2 (P62994), 14-3-3 protein zeta/delta (P63102), Unconventional myosin-Vb (P70569), Glutamate receptor ionotropic, NMDA 2B (Q00960), Ripk2 protein (Q3B7U0), Tubulin beta-3 chain (Q4QQS4), Transducin beta-like protein 3 (Q5U2W5), Nuclear factor of kappa light polypeptide gene enhancer in B-cells 2, p49/p100 (Q5U2Z4), Nuclear factor NF-kappa-B p105 subunit (Q63369), Von Hippel-Lindau disease tumor supressor (Q64259), Translation initiation factor eIF-2B subunit alpha (Q64270), Serine/threonine-protein kinase PAK 2 (Q64303), Polyglutamine-binding protein 1 (Q6PCT5), Cb1-727 (Q7TP21), Protein quaking (Q91XU1), E3 ubiquitin-protein ligase TRIM63 (Q91Z63), Epidermal growth factor receptor related protein (Q91Z63), Epidermal growth factor receptor related protein (Q9ESE0), Drebrin-like protein (Q9JHL4), Casein kinase 1 epsilon (Q9JJ76), and Inhibitor of nuclear factor kappa-B kinase subunit beta (Q9QY78). The access numbers in green nodes correspond to: 78 kDa glucose-regulated protein (P06761), 40S ribosomal protein S14 (P13471), Enoyl-CoA hydratase, mitochondrial (P14604), 60S ribosomal protein L6 (P21533), and 40S ribosomal protein S3 (P62909). The names of the proteins corresponding to each access number in the red nodes are listed in the Supplementary Table S2.

After chronic F exposure, totals of 699, 643, and 591 proteins were identified by mass spectrometry in the control, 10, and 50 ppm F groups, respectively. In the quantitative analysis of the 10 ppm F *vs*. control groups, 229 proteins with altered expression were observed (Supplementary Table S1), and the majority of these proteins were upregulated (143 proteins). For the 50 ppm F *vs*. control group comparison a total of 284 proteins with altered expression were identified (Supplementary Table S2), and the majority (270 proteins) presented downregulation. Totals of 220, 179 and 155 proteins were identified exclusively in the control, 10, and 50 ppm F groups, respectively (Supplementary Tables S3–S5). The functional classification according to the most affected biological processes is presented in Figs 1 and 2 for the comparisons of 10 ppm F *vs*. control and 50 ppm F *vs*. control, respectively. Exposure to 10 ppm F led to the most pronounced alterations, with changes in 43 functional categories (Fig. 1). Among them, the categories that presented the highest percentage of affected genes were the pyridine nucleotide metabolic process (41%), the carboxylic acid metabolic process (38%), the nicotinamide nucleotide metabolic process (36%), cellular component assembly (29%), myofibril assembly (24%), actomyosin structure organization (20%), and cytoskeleton organization (20%). Exposure to the highest F concentration affected 27 functional categories (Fig. 2). Among them, the categories with the highest percentage of associated genes affected were protein polymerization (33%), positive regulation of organelle organization (33%), actin filament organization (29%), the response to metal ions (23%), the response to inorganic substances (23%), the nicotinamide nucleotide metabolic process (19%), the purine ribonucleoside triphosphate metabolic process (18%) and regulation of translation (18%). Figures 3 and 4 show the subnetworks generated by JActive Modules for the comparisons of 10 ppm F *vs*. control and 50 ppm F *vs*. control, respectively. When the animals were exposed to 10 ppm F (Fig. 3), most of the proteins with altered expression interacted with com *Pleiotrophin* (P63090) and *Rab GDP dissociation inhibitor alpha* (P50398), while some of them interacted with *RNA-binding protein PNO1* (Q6VBQ8) and other ones with *Mitogen-activated protein kinase 14* (MAPK14; P70618). Exposure to the highest F concentration caused alterations in many proteins, generating a complex interaction subnetwork (Fig. 4). In this case, many of the proteins with altered expression interacted with partners involved in general and specialized signalling networks such as *Mitogen-activated protein kinase 3* (MAPK3; P21708), *Mothers against decapentaplegic homolog 2* (SMAD2; O70436), *14-3-3 Protein gamma* (P61983), *14-3-3 protein zeta/delta* (P63102), *Beta-arrestin-1* (P29066), *Casein kinase 1 epsilon* (Q9JJ76), *Serine/threonine-protein kinase* (PAK2; Q64303), *Tumour necrosis factor* (TNF; P16599), *Glutamate receptor ionotropic, NMDA 2B* (Q00960), *Growth factor receptor-bound protein 2* (GRB2; P62994), *Protein kinase C epsilon type* (KPCE; P09216), and *Receptor interacting kinase 2* (RIPK2; Q3B7U0).

## Discussion

Despite reported symptoms in cases of excessive F ingestion, such as nausea, vomiting, diarrhoea, and abdominal pain^38–40^, the mechanisms leading to these alterations in the GIT are largely unknown. To our knowledge, this study was the first to provide mechanistic insights on this topic, taking advantage of the combination of morphometric and proteomic analyses.

The evaluation of enteric neurons is considered an important analysis of pathological effects on the enteric ganglia with the aim of more precisely identifying and describing disorders related to enteric innervation. In the ENS, morphological changes in the myenteric plexus can induce motor disorders^41^, can be correlated with inflammatory conditions^42^, or can reflect physiological processes, such as aging^43^.

An increase in the release of neurotransmitters that stimulate muscle contraction such as SP^44^, or a decrease in the release of neurotransmitters that promote muscle relaxation, such as NO^45^, can consequently modify the behaviour of the intestinal smooth muscle contraction. Both events might have occurred in the present study since we observed a significant increase and a significant decrease, respectively, in the average values of the SP varicosity area (Table 4) and the nNOS-IR neuron area, for example (Table 3). As the enteric neurons have an essential role in the intestinal function, these alterations in their morphology and/or density observed in the present study could help to explain the intestinal discomfort and symptoms caused by F, which is also corroborated by the significant decrease in the area of HuC/D–IR cell body areas.

Some authors have described the correlation between an increase in F ingestion and elevated serum levels of NO with disturbances of NO metabolism in chicks^46^. Other important connections were presented between excessive F intake and NO production^47^ and increased NOS activity in the brain^48^, leading to alterations of some mechanisms, including glutamate reuptake and lipid peroxidation^49^. The latter is extremely important in the development of oxidative stress^50^ and degenerative diseases^51, 52^. Specially the oxidative stress seems to be the mechanism involved in the neurodegeneration caused by F, and it has been found as a consequence of F toxicity in the brain and also in the intestine^47, 48, 53^. Our results offer evidence of the correlation between F intake and NO production in the enteric innervation of the duodenum since we observed that F could affect nNOS-IR myenteric neurons at the morphometric and quantitative levels. The results observed in this study could be explained by an alteration of NO production or nNOS activity, which occurs in the CNS^47^, since F is also an enzyme inhibitor and its interaction with enzymes has been described as its main cellular toxic effect^54^. Another important effect of NO on the myenteric plexus is the induction of VIP release^55^. This VIP release by the myenteric varicosities, in turn, induces the production of NO by nNOS-IR neurons^56, 57^. This mechanism could explain the results in the present study, as a significant increase in the area of the VIP-IR varicosities was found for both F concentrations compared to the control, which could be an attempt to compensate for the reduction in NO production that might be characterized by a decrease in the area of the nNOS-IR neurons^30^. This result could also be explained by VIP action being complementary to NO in the control of gastrointestinal motility^58^ by interacting with NO in the inhibition of smooth muscle contraction of the intestinal wall^59, 60^.

Another possible explanation of our results lies in both plexi (myenteric and submucous) working synchronously during intestinal function^58^ and the morphological alterations observed in our study in the myenteric varicosities VIP-IR could also reflect a mechanism involving VIP production in the submucous plexus, where its physiological role is correlated with immunological functions^61^ and anti-inflammatory action^62^. Therefore, it is possible that F could affect VIP varicosities in the myenteric plexus through alterations in the submucous plexus as an part of inflammatory process caused by the ingestion of high F concentrations.

Associated with these important findings of the morphological analysis, the proteomic approach revealed that the group exposed to 10 ppm of F, compared with control, presented most of the proteins with downregulated expression interacting with *Pleiotrophin* (P63090). Among these proteins were *Fasciculation and elongation protein zeta-1* (P97577), *Protein disulfide-isomerase* (P04785)*, Guanine nucleotide-binding protein subunit beta-2-like 1* (P63245), *Glycerol-3-phosphate dehydrogenase [NAD*(+)*]-cytoplasmic* (O35077), and *Glutathione S-transferase P* (P04906). This connection is relevant to the present study since *Pleiotrophin* is a neurotrophic factor expressed during CNS development^63^; its upregulation promotes peripheral nerve regeneration^64^ and is also related to increased neuronal density with the aim of restoring brain function^65^. Its correlation with the CNS has also been described in other studies showing its involvement in cellular differentiation^66^ and proliferation^67^, as well as postsynaptic specialization^63^. Because this protein plays an important role in neuronal homeostasis in the CNS and peripheral nervous system, downregulation of its interaction partners could indicate that the concentration of 10 ppm of F in drinking water could have a similar toxic effect on the ENS although this F concentration did not cause alterations in the density of the general population or the nitrergic neurons of the duodenum myenteric plexus.

Other proteins with altered expression after chronic F exposure include the small guanosine triphosphatase family Ras-related in brain (GTPases Rab) family of proteins. Considered key regulators of intracellular membrane trafficking, Rabs change between an inactive GDP-bound form and an active GTP-bound form, and this latter form can recruit to membranes a different set of downstream effectors responsible for vesicle formation, movement, tethering and fusion^68^. In the 10 ppm F group (Fig. 3), Rab-1A (Q6NYB7) and Rab-8B (P70550) were downregulated while Rab-5B (A1L1J8) and Rab-8C were absent. The great numbers of downregulated proteins in the 10 ppm F group (or even absent) might be due to the absence of Rab-5 (B and C) proteins, which might have negatively regulated their transcription, since Rab-5 works as a meeting point between signalling and trafficking^68^. We could speculate that downregulation of Rab proteins after 10 ppm of F exposure could lead to impaired intracellular trafficking, causing an accumulation of proteins as neurotransmitters, which could help to explain the morphological alterations observed in the VIP-IR, CGRP-IR and SP-IR varicosities, as well as the increase in the thickness of the tunica muscularis in the 50 ppm F group. Interestingly, the expression of Rab proteins was also altered upon exposure to 50 ppm F, including *Ras-related protein Rab-1A* (Q6NYB7), *Ras-related protein Rab-1B* (P10536), *Ras-related protein Rab-3A* (P63012), *Protein Rab5b* (A1L1J8), *Protein Rab5c* (B0BNK1), *Ras-related protein Rab-8B* (P70550), (Rab-1A and the latter 3 proteins were also downregulated upon exposure to 10 ppm F), Ras-related protein Rab-10 (P35281), Ras-related protein Rab-14 (P61107) and Ras-related protein Rab-26 (P51156). Rab-10 interacts with *Melatonin receptor type 1A* (P49218) which likely mediates the circadian actions of melatonin. Another protein that was found to interact with the proteins identified in the present study was *Casein kinase 1 epsilon* (Q9JJ76), a Ser-Thr protein kinase that acts as a key regulator of the central clock in the suprachiasmatic nucleus^69^ and that is also involved in circadian regulation of gene expression^70^. Interestingly, a circadian rhythm for F has been described in humans, with the highest plasma F concentration found around midday and the lowest one in late afternoon^71^.

The process of protein polymerization was the most affected upon exposure to 50 ppm F (Fig. 2), with downregulation of different types of actin, such as *Actin, alpha skeletal muscle* (P68136), *Actin, alpha cardiac muscle 1* (P68035), and *Actin, cytoplasmic 2* (P63269), as well as tubulins, such as *Tubulin alpha-1A chain* (P68370), *Tubulin alpha-1A chain* (Q6P9V9), *Tubulin alpha-1C chain* (Q6AYZ1), *Tubulin alpha-3 chain* (Q68FR8), *Tubulin alpha-4A chain* (Q5XIF6), *Tubulin alpha-8 chain* (Q6AY56) and *Tubulin beta-2A chain* (P85108). Actin filament-binding proteins were also downregulated, such as *Annexin A2* (Q07936), *Tropomyosin alpha-1 chain* (P04692) and *Adenylyl cyclase-associated protein 1* (CAP-1; Q08163). The last one directly regulates filament dynamics and has been implicated in a number of complex developmental and morphological processes including mRNA localization and the establishment of cell polarity (UNIPROT).

In our network, CAP-1 interacted with *Debrin-like protein* (Q9JHL4) which plays a role in reorganization of the actin cytoskeleton, formation of cell projections in neuron morphogenesis, and synapse formation. In non-muscle cells, *Tropomyosin alpha-1 chain* is implicated in stabilizing cytoskeleton actin filaments. *Annexin A2* is a calcium-regulated membrane-binding protein that binds to actin filaments. Also downregulated by F, *Coronin-1A* (Q91ZN1) is involved in cytoskeleton organization and rearrangement in neuronal cells^72^, and *GTP-binding nuclear protein Ran* (RAN; P62828) contributes to nucleocytoplasmic transport, RNA export, chromatin condensation, and cell cycle control (UNIPROT). Downregulation of all of these key proteins might disturb cell signalling and important cellular processes such as protein synthesis, cell motility and vesicle trafficking leading to intracellular accumulation of proteins. This protein concentration could be related to the results observed here, such as increased thickness of the *tunica muscularis* and increased average values of the enteric neuron and varicosity areas, and it could possibly be related to enteric neurotransmission production and release.

Also involved in cellular transport, *Fasciculation and elongation protein zeta-1* (FEZ-1; P97577) was absent in the group exposed to 10 ppm of F and was downregulated by 50 ppm of F, compared with control. This protein is an adapter of the transport mediated by kinesins considered “motor” proteins, transporting not only molecules but also organelles through a process that is dependent on ATP and microtubules. Intracellular protein transport is essential for neuronal differentiation, gene expression and cytoskeleton rearrangement^73^. Supression of FEZ-1 by si-RNA affects mitochondrial motility and neuronal morphology^74^ in the CNS, and its downregulation observed after F exposure could affect enteric neurons by compromising the intracellular transport.

Many proteins with altered expression in the group exposed to 50 ppm of F interacted with proteins involved in signalling pathways such as *Beta-arrestin-1* (P29066), *Casein kinase 1 epsilon* (Q9JJ76), *Debrin-like protein* (Q9JHL4), *Growth factor receptor-bound protein 2* (GRB2) (P62994), *Glutamate receptor ionotropic, NMDA 2B* (Q00960), *Mitogen-activated protein kinase 3*–*Mapk3* (P21708), *Serine/threonine-protein kinase PAK 2* (Q64303), *Protein kinase C epsilon type (PKC)* (P09216), *Mothers against decapentaplegic homolog 2* (SMAD 2; O70436), *Tumour necrosis factor* (P16599), *E3 ubiquitin-protein ligase TRIM63* (Q91Z63) and *14-3-3 protein epsilon* (P62260). Most of these interaction proteins are involved with signalling pathways related to cytoskeleton organization while some of the proteins are related to the organization of lysosomes, nucleosomes, ribosomes, Golgi and endoplasmic reticulum to a lesser extent (UNIPROT).

*Phosphatidylethanolamine-binding protein 1* (PEBP1; P31044), a synaptic signalling protein, was also downregulated by 50 ppm of F in the present study. This molecule is downregulated in several neurological conditions such as Alzheimer’s^75^ and other memory-related disorders^76^. Its localization at the synapse suggests that it may be a critical regulator of neuronal survival^77^. Downregulation of PEBP1 could be associated with cognitive impairment which has been suggested to occur under exposure to high doses of F^78, 79^, despite this theory still being debated.

Organelle organization is another important process that can be correlated with our results in which two proteins, which were upregulated by 50 ppm of F, could be involved, namely *78 kDa glucose-regulated protein* (P06761) and *40S ribosomal protein S3* (P62909), which participate in the organization of the endoplasmic reticulum and ribosomes, respectively. Despite the upregulation of some proteins, most of them were downregulated after the 50 ppm F exposure, such as *Calmodulin* (P62161), *Caspase-3* (P55213), *Coronin-1A* (Q91ZN1), *Segment polarity protein dishevelled homolog* (DVL-1) (Q9WVB9), *Eukaryotic translation initiation factor 5A-1* (Q3T1J1), *Fasciculation and elongation protein zeta-1* (P97577), *Guanine nucleotide-binding protein subunit beta-2-like 1* (P63245), *Glutathione S-transferase P* (P04906), *Heat shock protein HSP 90-alpha* (P82995), *Nuclear distribution protein nudE-like 1Ndel1* (Q78PB6), *Phosphatidylethanolamine-binding protein 1* (P31044), *Peroxiredoxin-5 mitochondrial* (Q9R063), *Ras-related protein Rab-26* (P51156), *40S ribosomal protein S15a* (P62246), *ADP/ATP translocase 1* (Q05962), *Serotransferrin* (P12346), *Tropomyosin alpha-1 chain 9* (P04692), *Cytochrome b-c1 complex subunit 1mitochondrial* (Q68FY0), *Cytochrome b-c1 complex subunit 1 mitochondrial* (Q68FY0), *Transitional endoplasmic reticulum ATPase* (P46462) and *Vimentin* (P31000). Most of these downregulated proteins are involved in the organization of components of the cytoskeleton, while some of them are related to the organization of ribosomes, centrosomes, endoplasmic reticulum and Golgi. This outcome suggests impairment in the organization of these organelles due to high F exposure, especially organelles related to the cytoskeleton. Among the proteins downregulated by 50 ppm F are *40S ribosomal protein S25* (RPS25; P62853), *40S ribosomal protein S15a* (RPS15; P62246), and *40S ribosomal protein SA* (RPSSA; P38983). These ribosomal proteins are involved in protein translation and other multiple extraribosomal activities, such as DNA repair, cell death, inflammation, tumorigenesis, ribosome assembly and transcriptional regulation (Uniprot). Due to its role in the assembly, stability and maturation of the 40S ribosomal unit, downregulation of RPSSA might compromise protein synthesis, thus contributing to the reduced level of many proteins upon exposure to 50 ppm F.

In summary, chronic exposure to the highest F concentration promoted in the duodenums of rats a significant increase in the thickness of the *tunica muscularis* and alteration in the protein expression profile. This alteration was more pronounced than in previous proteomic studies that employed similar F concentrations and that evaluated other organs such as the kidney^80–82^ brain^83, 84^, and liver^26, 31^. This difference might have occurred because nearly 75% of ingested F is absorbed in the small intestine, especially in the proximal portions^4^. In addition, when F is ingested, the cells from the intestinal wall are directly exposed to higher F concentrations, which is different from other cell types distributed in several organs that will have contact only with absorbed F^85^. Upon exposure to the lowest F concentration, most of the proteins had upregulated expression (Table S1), while the opposite occurred for the highest F concentration (Table S2). All of the data presented suggest an increase in protein synthesis upon exposure to low F doses and impairment of protein synthesis when high F doses are ingested, suggesting important alterations in many cellular processes due to exposure to F. These alterations might help to explain the important gastrointestinal symptoms reported in cases of excessive F exposure, especially those involving the ENS, since the morphological alterations observed on enteric neurons present a pattern that is similar to enteric neuropathies caused by important pathologies that affect GIT function.

## Electronic supplementary material

Supplementary Information
