# Supplementary material for: Enteric innervation combined with proteomics for the evaluation of the effects of chronic fluoride exposure on the duodenum of rats
Source: Sci Rep. 2017 Apr 21;7:1070. doi: 10.1038/s41598-017-01090-y (PMC5430799; doi:10.1038/s41598-017-01090-y)
Supplement: Supplementary file 1 — Supplementary Information [file 41598_2017_1090_MOESM1_ESM.pdf]

Title

Enteric innervation combined with proteomics for the evaluation of the effects of chronic fluoride exposure on the duodenum of rats

Carina Guimarães de Souza Melo [1]

Juliana Vanessa Colombo Martins Perles [2]

Jacqueline Nelisis Zanoni [2]

Sara Raquel Garcia de Souza [2]

Erika Xavier dos Santos [2]

Aline de Lima Leite [1]

Alessandro Domingues Heubel[1]

Camila Oliveira e Souza [1]

Juliana Gadelha de Souza [1]

Marília Afonso Rabelo Buzalaf [1*]

[1] Department of Biological Sciences, Bauru School of Dentistry, University of São Paulo, Bauru, Brazil

[2] Department of Morphophysiological Sciences, State University of Maringá, Maringá, Brazil

[*] **Corresponding author**

Marília Afonso Rabelo Buzalaf

Alameda Octávio Pinheiro Brisolla, 9-75

Bauru-SP, Brazil, 17012-901

Phone: # 55 14 3235-8346

Email: mbuzalaf@fob.usp.br

Supplementary Table S1. Proteins identified with altered expression (upregulation and downregulation) in the duodenum of rats of the 10 ppm F Group. Animals exposed to 10 ppm F for 30 days in the drinking water.

Access Number	Gene name	Protein name description	Score	Ratio of the protein expression 10 ppm F Group : Control Group
D3ZP59	Non-identified	Uncharacterized protein	353.69	1.92
E9PT29	Ddx17	Protein Ddx17	434.38	1.72
A0A096MIX2	Ddx17	DEAD (Asp-Glu-Ala-Asp) box polypeptide 17, isoform CRA_a	512.19	1.69
M3ZCQ3	LOC100910765	LOC100910765	285.43	1.69
D3ZJF8	Fcgbp	Protein Fcgbp	294.05	1.69
Q62669	Hbb-b1	Protein Hbb-b1	285.43	1.64
P01946	Hba1	Hemoglobin subunit alpha-1/2	2052.37	1.61
P0CG51	Ubb	Polyubiquitin-B	481.18	1.59
Q62667	Mvp	Major vault protein	164.18	1.54
Q08290	Cnn1	Calponin-1	189.1	1.52
P52555	Erp29	Endoplasmic reticulum resident protein 29	174.5	1.49
Q4KLZ6	Dak	Bifunctional ATP-dependent dihydroxyacetone kinase/FAD-AMP lyase (cyclizing)	251.54	1.45
B0BMS8	Myl9	Myl9 protein	8936.01	1.43
F1LY34	Gm16519	Protein Gm16519	342.81	1.41
G3V8L3	Lmna	Lamin A, isoform CRA_b	146.38	1.41
Q5XIV1	Pgk2	Phosphoglycerate kinase	248.28	1.41
Q64122	Myl9	Myosin regulatory light polypeptide 9	9023.9	1.41
P18666	Myl12b	Myosin regulatory light chain 12B	2513.31	1.39
D4A8N3	RGD1563956	Protein RGD1563956	342.81	1.37
P23358	Rpl12	60S ribosomal protein L12	375.5	1.37
P48679	Lmna	Prelamin-A/C	151.71	1.37
C0JPT7	Flna	Filamin alpha	929.87	1.35
P07632	Sod1	Superoxide dismutase [Cu-Zn]	254.03	1.35
P13832	Rlc-a	Myosin regulatory light chain RLC-A	2476.81	1.35
P48037	Anxa6	Annexin A6	128.4	1.35

Q6IFW5	Krt12	Keratin, type I cytoskeletal 12	139.52	1.35
Q6IFV3	Krt15	Keratin, type I cytoskeletal 15	259.73	1.33
D3ZSM0	Non-identified	Uncharacterized protein	262.23	1.32
D4A931	Krt12	Keratin, type I cytoskeletal 12	139.52	1.32
M0R4B6	Rpl12	RCG64424	375.5	1.32
M0R6X5	Non-identified	Histone H2A	534.18	1.32
M0RCG4	Myl6l	Protein LOC100910474	1387.68	1.32
M0RDR9	Col6a1	Protein Col6a1	233.96	1.32
P10111	Ppia	Peptidyl-prolyl cis-trans isomerase A	7154.46	1.32
P31000	Vim	Vimentin	449.15	1.32
Q4V8H5	Dnpep	Aspartyl aminopeptidase	213.94	1.32
Q63279	Krt19	Keratin, type I cytoskeletal 19	4635.26	1.32
D3ZVT6	Asb7	Ankyrin repeat and SOCS box-containing 7	158.15	1.3
P48675	Des	Desmin	4826.38	1.3
Q00715	Non-identified	Histone H2B type 1	10186.13	1.3
Q6IFU7	Krt42	Keratin, type I cytoskeletal 42	744.07	1.3
Q6IFV1	Krt14	Keratin, type I cytoskeletal 14	750.39	1.3
Q6P725	Des	Desmin	4826.38	1.3
G3V9C7	Hist1h2bk	Histone H2B	10186.13	1.28
Q6IFU8	Krt17	Keratin, type I cytoskeletal 17	815.39	1.28
Q6IFV4	Krt13	Keratin, type I cytoskeletal 13	843.12	1.28
D3ZYW2	Hnrnph1	Heterogeneous nuclear ribonucleoprotein H	148.84	1.27
D4A817	LOC100912338	Histone H2B	10186.13	1.27
M0R5J4	Non-identified	Uncharacterized protein	434.13	1.27
P62963	Pfn1	Profilin-1	348.49	1.27
Q6AY07	Aldoart2	Fructose-bisphosphate aldolase	269.91	1.27
Q6P9V9	Tuba1b	Tubulin alpha-1B chain	2611.01	1.27
D4A115	Col6a3	Protein Col6a3	198.34	1.25
F1LNH3	Col6a2	Procollagen, type VI, alpha 2, isoform CRA_a	222.52	1.25
P06761	Hspa5	78 kDa glucose-regulated protein	1442.94	1.23
P68370	Tuba1a	Tubulin alpha-1A chain	2611.01	1.23
Q4QRB4	Tubb3	Tubulin beta-3 chain	2929.78	1.23
Q64119	Myl6	Myosin light polypeptide 6	1577.45	1.23

Q68FR8	Tuba3a	Tubulin alpha-3 chain	192.19	1.23
Q6AYZ1	Tuba1c	Tubulin alpha-1C chain	2648.47	1.23
D3ZEN5	Prdx5	Peroxiredoxin-5, mitochondrial	1092.29	1.22
M0R5B4	Non-identified	Uncharacterized protein	2432.08	1.22
M0RBL5	Non-identified	Uncharacterized protein	224.17	1.22
P00884	Aldob	Fructose-bisphosphate aldolase B	2972.1	1.22
P25030	Krt20	Keratin, type I cytoskeletal 20	1113.48	1.22
Q5BJY9	Krt18	Keratin, type I cytoskeletal 18	765.96	1.22
Q6P9T8	Tubb4b	Tubulin beta-4B chain	4837.4	1.22
Q9R063	Prdx5	Peroxiredoxin-5, mitochondrial	1092.29	1.22
D3ZD09	Cox6b1	Cytochrome c oxidase subunit 6B1	4235.68	1.2
E9PTU4	Myh11	Myosin-11	2660.33	1.2
P11598	Pdia3	Protein disulfide-isomerase A3	706.87	1.2
P13084	Npm1	Nucleophosmin	567.98	1.2
Q5XIF6	Tuba4a	Tubulin alpha-4A chain	238.76	1.2
Q6AZ25	Tpm1	Tropomyosin 1, alpha	1281.55	1.2
B5DFH4	Papss2	Papss2 protein	2030.74	1.19
D3ZLY9	Hist1h2bh	Histone H2B	10186.13	1.19
D3ZNZ9	Hist3h2ba	Histone H2B	10186.13	1.19
F1LPR6	Non-identified	Uncharacterized protein	3019.83	1.19
F1LZI1	LOC680121	Protein LOC680121	1185.85	1.19
P07335	Ckb	Creatine kinase B-type	2401.02	1.19
P46462	Vcp	Transitional endoplasmic reticulum ATPase	332.31	1.19
Q3KRE8	Tubb2b	Tubulin beta-2B chain	5228.32	1.19
Q63610	Tpm3	Tropomyosin alpha-3 chain	910.19	1.19
Q66HT1	Aldob	Fructose-bisphosphate aldolase	3339.1	1.19
D3Z7Y6	Krt20	Keratin, type I cytoskeletal 20	1113.48	1.18
D3ZIA7	Agr2	Anterior gradient 2 (Xenopus laevis) (Predicted), isoform CRA_a	2954.19	1.18
D4A4S3	Non-identified	Uncharacterized protein	399.57	1.18
G3V6D3	Atp5b	ATP synthase subunit beta	4833.17	1.18
G3V7C6	Tubb4b	RCG45400	5468.93	1.18
M0RBQ5	Hist3h2bb	Histone H2B	10186.13	1.18
M0RCB1	LOC102549957	Protein LOC102549957	2328.01	1.18

P55063	Hspa1l	Heat shock 70 kDa protein 1-like	1514.18	1.18
P69897	Tubb5	Tubulin beta-5 chain	5381.69	1.18
P85108	Tubb2a	Tubulin beta-2A chain	5257.43	1.18
D3ZNH4	Hist1h2bo	Histone H2B	10186.13	1.16
G3V6P7	LOC100911597	Myosin, heavy polypeptide 9, non-muscle	236.11	1.16
M0R8M9	Hspa8	Heat shock cognate 71 kDa protein	2699.48	1.16
P05065	Aldoa	Fructose-bisphosphate aldolase A	1056.3	1.16
P10719	Atp5b	ATP synthase subunit beta, mitochondrial	4833.17	1.16
P63018	Hspa8	Heat shock cognate 71 kDa protein	2676.97	1.16
Q10758	Krt8	Keratin, type II cytoskeletal 8	4242.51	1.16
Q63862	Myh11	Myosin-11	2051.26	1.16
Q68FY0	Uqcrc1	Cytochrome b-c1 complex subunit 1, mitochondrial	340.51	1.16
Q9ESV6	Gapdhs	Glyceraldehyde-3-phosphate dehydrogenase, testis-specific	3498.33	1.16
Q9Z1P2	Actn1	Alpha-actinin-1	505.14	1.16
F1M3U4	Non-identified	Uncharacterized protein	1314.16	1.15
M0RDZ9	Non-identified	Uncharacterized protein	2277.4	1.15
P02091	Hbb	Hemoglobin subunit beta-1	7606.81	1.15
P14659	Hspa2	Heat shock-related 70 kDa protein 2	1760.28	1.15
Q4QQV0	Tubb6	Protein Tubb6	2421.59	1.15
Q62812	Myh9	Myosin-9	222.3	1.15
P02770	Alb	Serum albumin	1709.88	1.14
P0DMW0	Hspa1a	Heat shock 70 kDa protein 1A	1514.18	1.14
B1WBQ8	Gapdhs	Glyceraldehyde-3-phosphate dehydrogenase	3498.33	1.12
B4F7C2	Tubb4a	Protein Tubb4a	3836.72	1.12
D4A111	Col6a3	Protein Col6a3	101.9	1.12
P04636	Mdh2	Malate dehydrogenase, mitochondrial	3826.73	1.12
Q9ER34	Aco2	Aconitate hydratase, mitochondrial	429.22	1.12
F7FK40	Tpm1	Tropomyosin 1, alpha, isoform CRA_c	2083.34	1.11
O88752	Hbe1	Epsilon 1 globin	4171.59	1.11
O88989	Mdh1	Malate dehydrogenase, cytoplasmic	896.63	1.11
P00770	Mcpt2	Mast cell protease 2	1459.96	1.11
P04692	Tpm1	Tropomyosin alpha-1 chain	1652.65	1.11
P11517	Non-identified	Hemoglobin subunit beta-2	4373.36	1.11
Q91XN6	Tpm1	Tropomyosin 1, alpha, isoform CRA_h	1652.65	1.11

Q9QXQ0	Actn4	Alpha-actinin-4	738.19	1.11
P05197	Eef2	Elongation factor 2	1019.12	1.1
D3ZGY4	Gapdh-ps2	Glyceraldehyde-3-phosphate dehydrogenase	5595.83	1.09
E9PTN6	RGD1564688	Glyceraldehyde-3-phosphate dehydrogenase	5588.86	1.09
F1LP05	Atp5a1	ATP synthase subunit alpha	3199.46	1.09
D3ZWM5	Hist1h2bb	Histone H2B	10186.13	1.08
M0R590	LOC685186	Glyceraldehyde-3-phosphate dehydrogenase	5595.83	1.08
P15999	Atp5a1	ATP synthase subunit alpha, mitochondrial	3209.54	1.08
P04797	Gapdh	Glyceraldehyde-3-phosphate dehydrogenase	5595.83	1.06
P62738	Acta2	Actin, aortic smooth muscle	15014.96	1.06
P63269	Actg2	Actin, gamma-enteric smooth muscle	14891.44	1.06
P68035	Actc1	Actin, alpha cardiac muscle 1	15846.68	1.06
D3ZRN3	Actbl2	Protein Actbl2	6740.97	1.05
P60711	Actb	Actin, cytoplasmic 1	19399.04	1.05
P68136	Acta1	Actin, alpha skeletal muscle	14594.31	1.05
P63259	Actg1	Actin, cytoplasmic 2	19399.04	1.04
V9GZ85	LOC100361457	Actin, cytoplasmic 2	19399.04	1.04
D3ZXS6	RGD1566344	Elongation factor 1-alpha	980.66	0.93
G3V9Y1	Myh10	Myosin, heavy polypeptide 10, non-muscle, isoform CRA_b	242.11	0.93
M0R757	LOC100360413	Elongation factor 1-alpha	980.66	0.93
P62630	Eef1a1	Elongation factor 1-alpha 1	980.66	0.93
M0RAS8	Non-identified	Elongation factor 1-alpha	980.66	0.93
P38983	Rpsa	40S ribosomal protein SA	1857.71	0.92
Q9JLT0	Myh10	Myosin-10	244.53	0.92
P04785	P4hb	Protein disulfide-isomerase	246.9	0.9
D3ZWE0	Hist2h2ab	Histone H2A	681.81	0.89
P04904	Gsta3	Glutathione S-transferase alpha-3	443.91	0.88
P38918	Akr7a3	Aflatoxin B1 aldehyde reductase member 3	1742.29	0.88
Q5BJT9	Ckmt1b	Creatine kinase, mitochondrial 1, ubiquitous	464.88	0.88
F1LML2	Ubc	Polyubiquitin-B	481.18	0.86
G3V9Z2	LOC100360645	Protein LOC100360645	481.18	0.86
Q07936	Anxa2	Annexin A2	457	0.86
M0RBL0	Akp3	Alkaline phosphatase	178.63	0.85
P04182	Oat	Ornithine aminotransferase, mitochondrial	658.6	0.85

M0R9W7	LOC679149	Carboxylic ester hydrolase	222	0.85
G3V8I8	Akp3	Alkaline phosphatase	178.63	0.84
P31232	Tagln	Transgelin	1819.18	0.84
G3V8A9	Pnliprp1	Triacylglycerol lipase	203.89	0.83
G3V9D8	Ces2c	Carboxylic ester hydrolase	135.77	0.83
P54316	Pnliprp1	Inactive pancreatic lipase-related protein 1	203.89	0.83
Q6NYB7	Rab1A	Ras-related protein Rab-1A	226.03	0.83
P04906	Gstp1	Glutathione S-transferase P	915.84	0.82
P07338	Ctrb1	Chymotrypsinogen B	2068.08	0.81
F1MA56	Ctrb1	Chymotrypsinogen B	2068.08	0.8
P00406	Mtco2	Cytochrome c oxidase subunit 2	708.6	0.79
P14942	Gsta4	Glutathione S-transferase alpha-4	357	0.79
P58775	Tpm2	Tropomyosin beta chain	1205.75	0.79
Q8SEZ5	Mt-co2	Cytochrome c oxidase subunit 2	708.6	0.78
D4ADS8	Rab4a	Ras-related protein Rab-4A	299.11	0.76
M0R6Y8	RGD1560402	Phosphoglycerate kinase	248.28	0.76
P55260	Anxa4	Annexin A4	288.42	0.76
P04642	Ldha	L-lactate dehydrogenase A chain	1875.49	0.76
P13221	Got1	Aspartate aminotransferase, cytoplasmic	143.63	0.76
G3V976	Cpa2	Carboxypeptidase A2	727.58	0.75
P05714	Rab4a	Ras-related protein Rab-4A	299.11	0.75
P19222	Cpa2	Carboxypeptidase A2	727.58	0.75
P63245	Gnb2l1	Guanine nucleotide-binding protein subunit beta-2-like 1	297.71	0.75
Q5RKJ9	Rab10	RAB10, member RAS oncogene family	226.03	0.75
F1M5M8	Rab37	Protein LOC100364984	226.03	0.74
P62804	Hist1h4b	Histone H4	5707.84	0.74
P63039	Hspd1	60 kDa heat shock protein, mitochondrial	257.82	0.74
Q5U362	Anxa4	Annexin	288.42	0.74
P10536	Rab1b	Ras-related protein Rab-1B	226.03	0.72
D4A0G7	Rab37	Protein LOC100364984	226.03	0.72
G3V6H0	LOC100363782	Protein LOC100363782	226.03	0.72
P51156	Rab26	Ras-related protein Rab-26	226.03	0.72
P70550	Rab8b	Ras-related protein Rab-8B	226.03	0.7
Q05962	Slc25a4	ADP/ATP translocase 1	716.67	0.68

Q09073	Slc25a5	ADP/ATP translocase 2	716.67	0.68
Q6P9Y4	Slc25a4	ADP/ATP translocase 1	469.81	0.68
E2RUH2	Rnh1	Ribonuclease inhibitor	208.05	0.67
Q8CJD3	Zg16	Zymogen granule membrane protein 16	404.48	0.65
P29315	Rnh1	Ribonuclease inhibitor	208.05	0.65
D3ZLL8	LOC691716	Protein LOC100909878	291.28	0.63
G3V741	Slc25a3	Phosphate carrier protein, mitochondrial	154.56	0.63
G3V7Q8	Prss3	Cationic trypsinogen	248.17	0.63
G3V8A7	Pnlip	Triacylglycerol lipase	1482.54	0.63
P08426	Try3	Cationic trypsin-3	248.17	0.63
P27657	Pnlip	Pancreatic triacylglycerol lipase	1482.54	0.63
G3V6S2	Aco1	Aconitate hydratase	199.01	0.63
P42123	Ldhb	L-lactate dehydrogenase B chain	921.55	0.63
D3ZAM3	Cpb1	Carboxypeptidase B	1290.17	0.62
M0RC99	Rab5a	Ras-related protein Rab-5A	120.27	0.61
P16036	Slc25a3	Phosphate carrier protein, mitochondrial	188.86	0.61
P19223	Cpb1	Carboxypeptidase B	1321.55	0.61
P62246	Rps15a	40S ribosomal protein S15a	291.28	0.6
E9PSI7	Amy2a3	Alpha-amylase	6772.62	0.58
P00689	Amy2	Pancreatic alpha-amylase	7901.7	0.58
P55091	Ctrc	Chymotrypsin-C	383.63	0.58
P07340	Atp1b1	Sodium/potassium-transporting ATPase subunit beta-1	484.95	0.56
A0A096MJI9	Atp1b1	Sodium/potassium-transporting ATPase subunit beta-1	484.95	0.55
G3V844	Amy2a3	Alpha-amylase	7901.27	0.55
P00773	Cela1	Chymotrypsin-like elastase family member 1	258.39	0.54
G3V7G2	Cel	Carboxylic ester hydrolase	388.06	0.53
P00762	Prss1	Anionic trypsin-1	457.37	0.51
P07882	Cel	Bile salt-activated lipase	388.06	0.51
P00774	Cela2a	Chymotrypsin-like elastase family member 2A	455.68	0.48
Q9Z1N4	Bpnt1	3'(2'),5'-bisphosphate nucleotidase 1	238.12	0.48
F1M219	Non-identified	Alpha-amylase	1433.91	0.48
P00731	Cpa1	Carboxypeptidase A1	135.64	0.48
E9PSQ1	Amy1a	Alpha-amylase	1384.24	0.46
G3V729	Prg2	Bone marrow proteoglycan	349.15	0.42

Q63189	Prg2	Bone marrow proteoglycan	349.15	0.39
G3V786	Akr1b8	Protein Akr1b8	248.15	0.37
Q5M875	Hsd17b13	17-beta-hydroxysteroid dehydrogenase 13	125.05	0.37

The proteins identified are organized in a decrescent order of the ratio expression (10 ppm F Group: Control Group). The identification was performed according to UNIPROT database (http://www.uniprot.org/).

Supplementary Table S2. Proteins identified with altered expression (upregulation and downregulation) in the duodenum of rats of the 50 ppm F Group. Animals exposed to 50 ppm F for 30 days in the drinking water.

**Access Number**	**Gene name**	**Protein name description**	**Score**	**Ratio of the protein expression 50 ppm F Group : Control Group**
F1M516	Non-identified	Uncharacterized protein	250	2.27
Q62669	Hbb-b1	Protein Hbb-b1	285	2.17
P0CG51	Ubb	Polyubiquitin-B	481	1.59
P00773	Cela1	Chymotrypsin-like elastase family member 1	258	1.56
P01946	Hba1	Hemoglobin subunit alpha-1/2	2052	1.32
P16409	Myl3	Myosin light chain 3	341	1.32
D3ZFG3	Cela3b	Elastase 3B, pancreatic, isoform CRA_b	304	1.3
Q00729	Hist1h2ba	Histone H2B type 1-A	320	1.26
D3ZSM0	Non-identified	Uncharacterized protein	262	1.2
P02770	Alb	Serum albumin	1710	1.15
D3ZJF8	Fcgbp	Protein Fcgbp	294	1.11
P06761	Hspa5	78 kDa glucose-regulated protein	1443	1.09
Q08290	Cnn1	Calponin-1	189	1.27
C0JPT7	Flna	Filamin alpha	930	0.94
M0RCB1	LOC102549957	Protein LOC102549957	2328	0.94
O88752	Hbe1	Epsilon 1 globin	4172	0.94
P02091	Hbb	Hemoglobin subunit beta-1	7607	0.94
F7FK40	Tpm1	Tropomyosin 1, alpha, isoform CRA_c	2083	0.93

M0R8M9	Hspa8	Heat shock cognate 71 kDa protein	2699	0.93
P63018	Hspa8	Heat shock cognate 71 kDa protein	2677	0.93
A0A0A0MXW3	H2afz	Histone H2A.Z	1030	0.91
D4ACV3	Hist2h2ac	Histone H2A	1030	0.91
F1LML2	Ubc	Polyubiquitin-B	481	0.91
P04692	Tpm1	Tropomyosin alpha-1 chain	1653	0.91
P0C169	Non-identified	Histone H2A type 1-C	1030	0.91
P13832	Rlc-a	Myosin regulatory light chain RLC-A	2477	0.91
P18666	Myl12b	Myosin regulatory light chain 12B	2513	0.91
Q63081	Pdia6	Protein disulfide-isomerase A6	356	0.91
A9UMV8	H2afj	Histone H2A.J	1030	0.90
D3ZWE0	Hist2h2ab	Histone H2A	682	0.90
D3ZXP3	H2afx	Histone H2A	1030	0.90
D4AEC0	H2afv	Histone H2A	1030	0.90
P02262	Non-identified	Histone H2A type 1	1030	0.90
P07338	Ctrb1	Chymotrypsinogen B	2068	0.90
P0C0S7	H2afz	Histone H2A.Z	1030	0.90
P0C170	Non-identified	Histone H2A type 1-E	1030	0.90
P0CC09	Hist2h2aa3	Histone H2A type 2-A	1030	0.90
Q4FZT6	Non-identified	Histone H2A type 3	1030	0.90
Q64598	Non-identified	Histone H2A type 1-F	1030	0.90
Q9QXQ0	Actn4	Alpha-actinin-4	738	0.90
Q9Z1P2	Actn1	Alpha-actinin-1	505	0.90
D3ZIA7	Agr2	Anterior gradient 2 (Xenopus laevis)	2954	0.90
D3ZVK7	Hist1h2ak	Histone H2A	1030	0.90
M0RCL5	LOC100910554	Histone H2A	1030	0.90
M0RDM4	LOC680322	Histone H2A	1030	0.90
Q6I8Q6	Hist1h2af	Histone H2A	1030	0.90
P07335	Ckb	Creatine kinase B-type	2401	0.89
P48675	Des	Desmin	4826	0.89
Q00728	Non-identified	Histone H2A type 4	1030	0.89
Q6P725	Des	Desmin	4826	0.89
D4A115	Col6a3	Protein Col6a3	198	0.88
D4A111	Col6a3	Protein Col6a3	102	0.87
F1LNH3	Col6a2	Procollagen, type VI, alpha 2, isoform CRA_a	223	0.87

E9PTU4	Myh11	Myosin-11	2660	0.86
G3V9Z2	LOC100360645	Protein LOC100360645	481	0.86
D3ZUL3	Col6a1	Protein Col6a1	476	0.84
P82995	Hsp90aa1	Heat shock protein HSP 90-alpha	1114	0.84
Q6IFV4	Krt13	Keratin, type I cytoskeletal 13	843	0.84
M0R6J6	Ckmt2	Creatine kinase S-type, mitochondrial	322	0.84
M0R6X5	Non-identified	Histone H2A	534	0.84
P09605	Ckmt2	Creatine kinase S-type, mitochondrial	322	0.84
P10719	Atp5b	ATP synthase subunit beta, mitochondrial	4833	0.84
Q63862	Myh11	Myosin-11	2051	0.84
G3V6D3	Atp5b	ATP synthase subunit beta	4833	0.83
Q9ER34	Aco2	Aconitate hydratase, mitochondrial	429	0.83
B5DFH4	Papss2	Papss2 protein	2031	0.82
F1M6C2	LOC100360150	Elongation factor 1-alpha	750	0.82
G3V8C3	Vim	Vimentin	442	0.82
P18418	Calr	Calreticulin	413	0.82
P34058	Hsp90ab1	Heat shock protein HSP 90-beta	2306	0.82
Q09073	Slc25a5	ADP/ATP translocase 2	717	0.82
D3ZXS6	RGD1566344	Elongation factor 1-alpha	981	0.81
G3V8A9	Pnliprp1	Inactive pancreatic lipase-related protein 1	204	0.81
G3V9D8	Ces2c	Carboxylic ester hydrolase	136	0.81
M0R757	LOC100360413	Elongation factor 1-alpha	981	0.81
M0RAS8	Non-identified	Elongation factor 1-alpha	981	0.81
M0RBL5	Non-identified	Uncharacterized protein	224	0.81
P05065	Aldoa	Fructose-bisphosphate aldolase A	1056	0.81
P31000	Vim	Vimentin	449	0.81
P61107	Rab14	Ras-related protein Rab-14	226	0.81
P62630	Eef1a1	Elongation factor 1-alpha 1	981	0.81
P62632	Eef1a2	Elongation factor 1-alpha 2	750	0.81
G3V976	Cpa2	Carboxypeptidase A2	728	0.80
M0R8B6	Tubb1	Protein Tubb1	679	0.80
P54316	Pnliprp1	Inactive pancreatic lipase-related protein 1	204	0.80
P62804	Hist1h4b	Histone H4	5708	0.80
Q5D059	LOC100363335	Hnrpk protein	261	0.80
F1LTA7	LOC100909504	Protein LOC100909504	2181	0.79

P19222	Cpa2	Carboxypeptidase A2	728	0.79
P25809	Ckmt1	Creatine kinase U-type, mitochondrial	232	0.79
Q6P3V8	Eif4a1	Eukaryotic translation initiation factor 4A1	258	0.79
R9PXU6	Vcl	Vinculin	218	0.79
F1LPR6	Ighm	Uncharacterized protein	3020	0.79
F1LTQ2	Non-identified	Uncharacterized protein	6608	0.79
P11884	Aldh2	Aldehyde dehydrogenase, mitochondrial	288	0.79
P38918	Akr7a3	Aflatoxin B1 aldehyde reductase member 3	1742	0.79
P62824	Rab3c	Ras-related protein Rab-3C	177	0.79
Q6AY07	Aldoart2	Fructose-bisphosphate aldolase	270	0.79
D3ZYW2	Hnrnph1	Heterogeneous nuclear ribonucleoprotein H	149	0.78
M0R9W7	LOC679149	Carboxylic ester hydrolase	222	0.78
P12346	Tf	Serotransferrin	572	0.78
Q7TMC7	Tf	Ab2-417	578	0.78
E9PU16	Rab1a	Ras-related protein Rab-1A	218	0.77
F1LN88	Aldh2	Aldehyde dehydrogenase, mitochondrial	285	0.77
O70177	LOC100365112	Carboxylesterase	189	0.77
P85972	Vcl	Vinculin	218	0.77
Q4QQV0	Tubb6	Protein Tubb6	2422	0.77
Q68FY0	Uqcrc1	Cytochrome b-c1 complex subunit 1, mitochondrial	341	0.77
P00884	Aldob	Fructose-bisphosphate aldolase B	2972	0.76
P55260	Anxa4	Annexin A4	288	0.76
P85973	Pnp	Purine nucleoside phosphorylase	525	0.76
Q4QRB4	Tubb3	Tubulin beta-3 chain	2930	0.76
D3ZXK9	Pnp	Purine nucleoside phosphorylase	525	0.76
M0RBY5	Non-identified	Uncharacterized protein	903	0.76
P04636	Mdh2	Malate dehydrogenase, mitochondrial	3827	0.76
P05714	Rab4a	Ras-related protein Rab-4A	299	0.76
Q68FR8	Tuba3a	Tubulin alpha-3 chain	192	0.76
A0A0A0MY09	Hsp90b1	Endoplasmin	554	0.75
D4ADS8	Rab4a	Ras-related protein Rab-4A	299	0.75
E9PSI7	Amy2a3	Alpha-amylase	6773	0.75
G3V9Q3	Hnrnph1	Heterogeneous nuclear ribonucleoprotein H	149	0.75
P14668	Anxa5	Annexin A5	154	0.75
P29315	Rnh1	Ribonuclease inhibitor	208	0.75

Q5BJT9	Ckmt1b	Creatine kinase, mitochondrial 1, ubiquitous	465	0.75
Q5U206	Calml3	Calmodulin-like protein 3	358	0.75
Q5U362	Anxa4	Annexin	288	0.75
Q5XIF6	Tuba4a	Tubulin alpha-4A chain	239	0.75
Q66HH8	Anxa5	Annexin	154	0.75
Q6AY56	Tuba8	Tubulin alpha-8 chain	179	0.75
E2RUH2	Rnh1	Ribonuclease inhibitor	208	0.74
G3V7J5	Ces2e	Carboxylesterase 5, isoform CRA_a	347	0.74
P00689	Amy2	Pancreatic alpha-amylase	7902	0.74
Q66HD0	Hsp90b1	Endoplasmin	558	0.74
D3ZVT6	Asb7	Ankyrin repeat and SOCS box-containing 7	158	0.73
G3V8A7	Pnlip	Pancreatic lipase, isoform CRA_a	1483	0.73
P27657	Pnlip	Pancreatic triacylglycerol lipase	1483	0.73
P46462	Vcp	Transitional endoplasmic reticulum ATPase	332	0.73
P85108	Tubb2a	Tubulin beta-2A chain	5257	0.73
Q07936	Anxa2	Annexin A2	457	0.73
Q66HT1	Aldob	Fructose-bisphosphate aldolase	3339	0.73
F7F3M3	Ces2a	Protein Ces2a	347	0.73
P63039	Hspd1	60 kDa heat shock protein, mitochondrial	258	0.73
P69897	Tubb5	Tubulin beta-5 chain	5382	0.73
Q3KRE8	Tubb2b	Tubulin beta-2B chain	5228	0.73
Q6NYB7	Rab1A	Ras-related protein Rab-1A	226	0.73
Q6P9Y4	Slc25a4	ADP/ATP translocase 1	470	0.73
D3Z7Y6	Krt20	Keratin, type I cytoskeletal 20	1113	0.72
D3ZXQ0	Ces2g	Protein Ces2g	347	0.72
Q05962	Slc25a4	ADP/ATP translocase 1	482	0.72
B5DFA0	Vil1	Protein Vil1	612	0.71
G3V6P7	Myh9	Myosin, heavy polypeptide 9, non-muscle	236	0.71
P04182	Oat	Ornithine aminotransferase, mitochondrial	659	0.71
P15999	Atp5a1	ATP synthase subunit alpha, mitochondrial	3210	0.71
P16617	Pgk1	Phosphoglycerate kinase 1	790	0.71
P63245	Gnb2l1	Guanine nucleotide-binding protein subunit beta-2-like 1	298	0.71
Q62812	Myh9	Myosin-9	222	0.71
Q63716	Prdx1	Peroxiredoxin-1	825	0.71
D3ZB30	Ptbp1	Polypyrimidine tract binding protein 1, isoform CRA_c	130	0.70

D3ZRN3	Actbl2	Protein Actbl2	6741	0.70
F1LP05	Atp5a1	ATP synthase subunit alpha	3199	0.70
F1M5M8	Rab37	Protein LOC100364984	226	0.70
G3V8L1	Pycard	PYD and CARD domain containing	389	0.70
P06768	Rbp2	Retinol-binding protein 2	552	0.70
P07756	Cps1	Carbamoyl-phosphate synthase [ammonia], mitochondrial	135	0.70
P35280	Rab8a	Ras-related protein Rab-8A	218	0.70
P68035	Actc1	Actin, alpha cardiac muscle 1	15847	0.70
P68136	Acta1	Actin, alpha skeletal muscle	14594	0.70
D4A5P1	Non-identified	Uncharacterized protein	3139	0.70
P38983	Rpsa	40S ribosomal protein SA	1858	0.70
P62738	Acta2	Actin, aortic smooth muscle	15015	0.70
P63269	Actg2	Actin, gamma-enteric smooth muscle	14891	0.70
Q5RKJ9	Rab10	RAB10, member RAS oncogene family	226	0.70
D4A0G7	Rab37	Protein LOC100364984	226	0.69
F1LM18	Ptbp1	Polypyrimidine tract-binding protein 1	130	0.69
P10536	Rab1b	Ras-related protein Rab-1B	226	0.69
P25030	Krt20	Keratin, type I cytoskeletal 20	1113	0.69
P35284	Rab12	Ras-related protein Rab-12	218	0.69
P63012	Rab3a	Ras-related protein Rab-3A	218	0.69
Q00438	Ptbp1	Polypyrimidine tract-binding protein 1	130	0.69
Q63941	Rab3b	Ras-related protein Rab-3B	244	0.69
P07943	Akr1b1	Aldose reductase	152	0.68
P51156	Rab26	Ras-related protein Rab-26	226	0.68
Q53B90	Rab43	Ras-related protein Rab-43	218	0.68
G3V7Q8	Prss3	Cationic trypsinogen	248	0.68
M0R4D7	LOC100910820	Protein LOC100910820	1986	0.68
P35281	Rab10	Ras-related protein Rab-10	218	0.68
P60711	Actb	Actin, cytoplasmic 1	19399	0.68
P63259	Actg1	Actin, cytoplasmic 2	19399	0.68
D3ZAU6	RGD1561919	Protein RGD1561919	205	0.67
P08426	Try3	Cationic trypsin-3	248	0.67
P19629	Ldhc	L-lactate dehydrogenase C chain	215	0.67
P51146	Rab4b	Ras-related protein Rab-4B	218	0.67
P70550	Rab8b	Ras-related protein Rab-8B	226	0.67

V9GZ85	LOC100361457	Actin, cytoplasmic 2	19399	0.67
B4F7C2	Tubb4a	Protein Tubb4a	3837	0.66
G3V6H0	LOC100363782	Protein LOC100363782	226	0.66
P06687	Atp1a3	Sodium/potassium-transporting ATPase subunit alpha-3	193	0.66
D3ZSY4	Epx	Eosinophil peroxidase	343	0.66
Q6AYX2	Ldhc	L-lactate dehydrogenase	215	0.66
D3ZCV0	Actn2	Protein Actn2	196	0.65
D3ZE29	Non-identified	Uncharacterized protein	252	0.65
D4AC62	Krt222	Protein Krt222	564	0.65
M0RDI1	LOC102550391	Glutathione S-transferase	444	0.65
D4A376	Rab12	Ras-related protein Rab-12	218	0.64
P62161	Calm1	Calmodulin	332	0.64
G3V8I1	Alpi	Alkaline phosphatase	415	0.64
G3V9Y1	Myh10	Myosin, heavy polypeptide 10, non-muscle, isoform CRA_b	242	0.64
Q9JLT0	Myh10	Myosin-10	245	0.64
M0RBL0	Akp3	Alkaline phosphatase	179	0.63
P00770	Mcpt2	Mast cell protease 2	1460	0.63
P06685	Atp1a1	Sodium/potassium-transporting ATPase subunit alpha-1	551	0.63
P19223	Cpb1	Carboxypeptidase B	1322	0.62
P50399	Gdi2	Rab GDP dissociation inhibitor beta	609	0.62
Q08163	Cap1	Adenylyl cyclase-associated protein 1	304	0.62
Q8VHF5	Cs	Citrate synthase, mitochondrial	270	0.62
G3V936	Cs	Citrate synthase	270	0.61
M0R451	Non-identified	Glyceraldehyde-3-phosphate dehydrogenase	2197	0.61
P50398	Gdi1	Rab GDP dissociation inhibitor alpha	430	0.61
Q8CJD3	Zg16	Zymogen granule membrane protein 16	404	0.61
F7EPH4	Ppa1	Protein Ppa1	162	0.61
G3V8I8	Akp3	Alkaline phosphatase	179	0.61
Q9ESV6	Gapdhs	Glyceraldehyde-3-phosphate dehydrogenase, testis-specific	3498	0.61
D4A3W5	Non-identified	Glyceraldehyde-3-phosphate dehydrogenase	2138	0.60
P04642	Ldha	L-lactate dehydrogenase A chain	1875	0.60
P38552	Lgals4	Galectin-4	519	0.60
Q63942	Rab3d	GTP-binding protein Rab-3D	157	0.60
D3ZAM3	Cpb1	Carboxypeptidase B	1290	0.59
G3V844	Amy2a3	Alpha-amylase	7901	0.59

P04906	Gstp1	Glutathione S-transferase P	916	0.59
B1WBQ8	Gapdhs	Glyceraldehyde-3-phosphate dehydrogenase	3498	0.59
F1M2N4	Non-identified	Uncharacterized protein	2197	0.59
M0R590	LOC685186	Glyceraldehyde-3-phosphate dehydrogenase	5596	0.59
P06686	Atp1a2	Sodium/potassium-transporting ATPase subunit alpha-2	193	0.58
E9PTN6	RGD1564688	Glyceraldehyde-3-phosphate dehydrogenase	5589	0.58
F1M779	Cltc	Clathrin heavy chain	143	0.58
G3V7C6	Tubb4b	RCG45400	5469	0.58
P11442	Cltc	Clathrin heavy chain 1	146	0.58
F1M219	Non-identified	Alpha-amylase	1434	0.57
P04797	Gapdh	Glyceraldehyde-3-phosphate dehydrogenase	5596	0.57
Q6P9V9	Tuba1b	Tubulin alpha-1B chain	2611	0.57
G3V741	Slc25a3	Phosphate carrier protein, mitochondrial	155	0.57
P55091	Ctrc	Chymotrypsin-C	384	0.57
P00406	Mtco2	Cytochrome c oxidase subunit 2	709	0.56
P16036	Slc25a3	Phosphate carrier protein, mitochondrial	189	0.56
D3ZGY4	Gapdh-ps2	Glyceraldehyde-3-phosphate dehydrogenase	5596	0.55
P62963	Pfn1	Profilin-1	348	0.54
Q8SEZ5	Mt-co2	Cytochrome c oxidase subunit 2	709	0.54
P04903	Gsta2	Glutathione S-transferase alpha-2	444	0.54
P67779	Phb	Prohibitin	250	0.54
M0R660	RGD1565368	Glyceraldehyde-3-phosphate dehydrogenase	5589	0.53
P50137	Tkt	Transketolase	342	0.53
P04904	Gsta3	Glutathione S-transferase alpha-3	444	0.52
F7F2H5	Gsta2	Glutathione S-transferase	444	0.52
P00502	Gsta1	Glutathione S-transferase alpha-1	444	0.52
G3V826	Tkt	Transketolase	279	0.51
P68370	Tuba1a	Tubulin alpha-1A chain	2611	0.50
Q4FZZ3	Gsta5	Glutathione S-transferase	444	0.50
B0BNA5	Cotl1	Coactosin-like protein	353	0.49
Q6AYZ1	Tuba1c	Tubulin alpha-1C chain	2648	0.49
E9PSQ1	Amy1a	Alpha-amylase	1384	0.48
Q6P9T8	Tubb4b	Tubulin beta-4B chain	4837	0.48

G3V7G2	Cel	Bile salt-activated lipase	388	0.47
P07882	Cel	Bile salt-activated lipase	388	0.47
D3Z8U8	Non-identified	Uncharacterized protein	2138	0.46
D3ZIY0	Non-identified	Uncharacterized protein	2138	0.46
P42123	Ldhb	L-lactate dehydrogenase B chain	922	0.46
M0R5B4	Non-identified	Uncharacterized protein	2432	0.46
M0RBH6	Non-identified	Uncharacterized protein	2138	0.46
E9PTV9	RGD1562758	Glyceraldehyde-3-phosphate dehydrogenase	2145	0.45
P00762	Prss1	Anionic trypsin-1	457	0.45
D3ZXS2	Non-identified	Uncharacterized protein	2138	0.44
Q68FR6	Eef1g	Elongation factor 1-gamma	133	0.44
F1M7P4	Prph	Peripherin	402	0.44
F1M9J9	Non-identified	Uncharacterized protein	2138	0.44
F1M4G6	Non-identified	Uncharacterized protein	2138	0.43
P21807	Prph	Peripherin	402	0.43
Q6IG12	Krt7	Keratin, type II cytoskeletal 7	323	0.42
G3V712	Krt7	Keratin complex 2, basic, gene 7, isoform CRA_a	323	0.41
P04166	Cyb5b	Cytochrome b5 type B	566	0.39
F1M3U4	Gm15294	Uncharacterized protein	1314	0.39
P07340	Atp1b1	Sodium/potassium-transporting ATPase subunit beta-1	485	0.35
G3V729	Prg2	Bone marrow proteoglycan	349	0.35
Q63189	Prg2	Bone marrow proteoglycan	349	0.34
A0A096MJI9	Atp1b1	Sodium/potassium-transporting ATPase subunit beta-1	485	0.34

The proteins identified are organized in a decrescent order of the ratio expression (50 ppm F Group: Control Group). The identification was performed according to UNIPROT database (http://www.uniprot.org/).

Supplementary Table S3. Proteins identified exclusively in the rats duodenum of the control group after chronic F exposure (ingestion of deionized water for 30 days).

Access Number	Gene name	Protein name description	Score
F1M6F4	LOC100912210	Protein LOC100912210	1335
M0R763	LOC100911337	Protein LOC100911337	1294
P62853	Rps25	40S ribosomal protein S25	1294
P00330	ADH1	Alcohol dehydrogenase 1	1009
D3ZAF6	Atp5j2	ATP synthase subunit f, mitochondrial	775
P10860	Glud1	Glutamate dehydrogenase 1, mitochondrial	603
P04166	Cyb5b	Cytochrome b5 type B	566
B2RZD6	Ndufa4	Ndufa4 protein	502
B2RZD1	Sec61b	Protein Sec61b	467
F7FLF2	Rpl22	Protein LOC100360057	463
P47198	Rpl22	60S ribosomal protein L22	463
B2RZ72	Arpc4	Actin related protein 2/3 complex, subunit 4 (Predicted), isoform CRA_a	452
B2RZA9	Ube2l3	Protein Ube2l3	422
G3V8L1	Pycard	PYD and CARD domain containing	389
G3V6G1	Igj	Immunoglobulin joining chain	383
P08010	Gstm2	Glutathione S-transferase Mu 2	376
Q5U206	Calml3	Calmodulin-like protein 3	358
D3Z8N2	Rnf187	E3 ubiquitin-protein ligase RNF187	357
D3ZXQ0	Ces2g	Protein Ces2g	347
F7F3M3	Ces2a	Protein Ces2a	347
G3V7J5	Ces2e	Carboxylesterase 5, isoform CRA_a	347
D4AB87	Gstm6l	Protein Gstm6l	328
Q00729	Hist1h2ba	Histone H2B type 1-A	320
P0C5J3	B9d2	B9 domain-containing protein 2	316
D3ZG05	LOC100912049	40S ribosomal protein S12	285
D3ZVQ8	Gstm6	Protein Gstm6	283

F1LXL7	Gstm6l	Protein Gstm6	283
Q5BK56	Gstm4	Glutathione S-transferase mu 4	283
Q9Z1B2	Gstm5	Glutathione S-transferase Mu 5	283
A0A096MJL6	Pgk1	Phosphoglycerate kinase 1	282
D3ZN21	Ddx3y	Protein Ddx3y	280
D4ADE8	Ddx3x	DEAD/H (Asp-Glu-Ala-Asp/His) box polypeptide 3, X-linked	280
Q5M860	Arhgdib	Protein Arhgdib	254
F1M516	Non-identified	Uncharacterized protein	250
Q91ZN1	Coro1a	Coronin-1A	247
P51635	Akr1a1	Alcohol dehydrogenase [NADP(+)]	240
P25235	Rpn2	Dolichyl-diphosphooligosaccharide--protein glycosyltransferase subunit 2	229
Q9WVB9	Dvl1	Segment polarity protein dishevelled homolog DVL-1	224
D4A376	Rab12	Ras-related protein Rab-12	218
E9PU16	Rab1a	Ras-related protein Rab-1A	218
P35280	Rab8a	Ras-related protein Rab-8A	218
P35281	Rab10	Ras-related protein Rab-10	218
P35284	Rab12	Ras-related protein Rab-12	218
P51146	Rab4b	Ras-related protein Rab-4B	218
P63012	Rab3a	Ras-related protein Rab-3A	218
Q53B90	Rab43	Ras-related protein Rab-43	218
Q5U316	Rab35	Ras-related protein Rab-35	218
D3ZX74	Coq10a	Coenzyme Q10 homolog A (Yeast) (Predicted), isoform CRA_b	215
D3ZD94	RGD1562107	Glutathione S-transferase	212
F1LVC6	LOC100365881	Glutathione S-transferase	212
F1M0Q4	LOC679594	Protein LOC679594	211
D3ZEZ9	Svil	Protein Svil	209
Q561S0	Ndufa10	NADH dehydrogenase [ubiquinone] 1 alpha subcomplex subunit 10, mitochondrial	207
F1M9K9	Kif1b	6-phosphogluconate dehydrogenase, decarboxylating	206
P85968	Pgd	6-phosphogluconate dehydrogenase, decarboxylating	206

D3ZAU6	RGD1561919	Protein RGD1561919	205
A0A096P6M0	Oaz3	Ornithine decarboxylase antizyme 3	205
A1BPI0	Oaz3	Ornithine decarboxylase antizyme 3	205
Q5RJR8	Lrrc59	Leucine-rich repeat-containing protein 59	205
P55213	Casp3	Caspase-3	201
D3ZFH6	Phb-ps1	RCG33110	201
F1M155	Svil	Protein Svil	201
D4AA19	RGD1306519	Uncharacterized protein	200
F1LWV4	RGD1306519	Uncharacterized protein	199
F1M7S2	Psmf1	Proteasome inhibitor PI31 subunit	196
Q5XIU5	Psmf1	Proteasome inhibitor PI31 subunit	196
Q925D2	Bik	Bcl2-interacting killer, isoform CRA_a	196
Q78PB6	Ndel1	Nuclear distribution protein nudE-like 1	195
D3ZQS5	Svil	Protein Svil	191
D4A8G5	Tgfbi	Protein Tgfbi	189
A1L128	Adh4	Alcohol dehydrogenase 4	189
Q64563	Adh4	Alcohol dehydrogenase 4	189
F1LU96	Nckap5	Protein Nckap5	185
M0RAD4	Nckap5	Protein Nckap5	185
F1M7S7	Rab17	Protein Rab17	184
D3ZF34	Non-identified	Uncharacterized protein	181
D3ZME6	Syncrip	Uncharacterized protein	181
Q68A21	Purb	Transcriptional activator protein Pur-beta	179
A1L1J8	Rab5b	Protein Rab5b	179
B0BNK1	Rab5c	Protein Rab5c	179
P35171	Cox7a2	Cytochrome c oxidase subunit 7A2, mitochondrial	179
D3ZUQ5	Vom2r34	Protein Vom2r35	179
F8WFF9	Lrrc57	Protein LOC100910478	178
P62824	Rab3c	Ras-related protein Rab-3C	177
F1LQS6	Xdh	RCG61833	177
P22985	Xdh	Xanthine dehydrogenase/oxidase	177
Q499N1	Rara	Protein Rara	171
M0R5T0	Nckap5	Protein Nckap5	168
D4ADU8	Non-identified	Pyruvate kinase	167
D3ZIE1	RGD1566369	40S ribosomal protein S8	164

F1LSQ6	Psma7	Proteasome subunit alpha type	163
F1M6I7	Psma8	Proteasome subunit alpha type	163
D4AEL0	LOC691083	Protein LOC691083	162
M0R7B5	LOC102554611	Protein LOC102554611	161
Q68FU3	Etfb	Electron transfer flavoprotein subunit beta	161
Q9WVJ6	Tgm2	Protein Tgm2	160
D3ZLP4	Tuba1b	Uncharacterized protein	160
F1LRJ9	Selenbp1	Uncharacterized protein	159
Q8VIF7	Selenbp1	Selenium-binding protein 1	159
Q63942	Rab3d	GTP-binding protein Rab-3D	157
F1LXU3	Gpr87	Protein Gpr87	157
P21643	Tdo2	Tryptophan 2,3-dioxygenase	153
P07943	Akr1b1	Aldose reductase	152
P32551	Uqcrc2	Cytochrome b-c1 complex subunit 2, mitochondrial	151
D3ZEV0	LOC100912427	Protein LOC100912427	150
F1LPL7	LOC100912427	Protein LOC100912427	150
P62961	Ybx1	Nuclease-sensitive element-binding protein 1	150
Q3ZAV2	Ybx1	Uncharacterized protein	150
B0BNJ1	Sri	LOC683667 protein	148
D3Z860	Btbd19	Protein Btbd19	148
O35152	Bet1l	BET1-like protein	147
M0R3K9	LOC365828	Protein LOC365828	146
Q498S5	Pex7	Peroxisomal biogenesis factor 7	146
P11442	Cltc	Clathrin heavy chain 1	146
Q8VHV7	Hnrnph1	Heterogeneous nuclear ribonucleoprotein H	146
F1M779	Cltc	Clathrin heavy chain	143
D4AD84	Non-identified	Uncharacterized protein	142
Q5XI78	Ogdh	2-oxoglutarate dehydrogenase, mitochondrial	142
Q6AXX2	Non-identified	Uncharacterized protein C16orf46 homolog	141
D3ZAN3	Ganab	Alpha glucosidase 2 alpha neutral subunit	140
D3ZKR3	Non-identified	Glyceraldehyde-3-phosphate dehydrogenase	140
F1LTU2	Non-identified	Glyceraldehyde-3-phosphate dehydrogenase	140
B5DEN5	Eef1b2	Eukaryotic translation elongation factor 1 beta 2	140
F1M0U2	Non-identified	Uncharacterized protein	140
D4A1W8	Mttp	Microsomal triglyceride transfer protein	139

F1LR22	Smyd3	Protein Smyd3	138
Q3T1J1	Eif5a	Eukaryotic translation initiation factor 5A-1	138
P80385	Prkag1	5'-AMP-activated protein kinase subunit gamma-1	138
D4A7P2	Lrrtm2	Leucine-rich repeat transmembrane neuronal protein 2	137
M0RC63	Lrrtm2	Leucine-rich repeat transmembrane neuronal protein 2	137
G3V9J5	Styxl1	Protein Styxl1	137
P0C219	Slmap	Sarcolemmal membrane-associated protein	136
G3V8S4	Atp12a	ATPase, H+/K+ transporting, nongastric, alpha polypeptide, isoform CRA_a	136
P54708	Atp12a	Potassium-transporting ATPase alpha chain 2	136
D4ABX0	Dcaf12l1	Protein Dcaf12l1	136
M0R4D8	Non-identified	Uncharacterized protein	133
M0RB43	Non-identified	Uncharacterized protein	133
D3ZZ81	Ppfia1	Protein Ppfia1	132
F1LWN5	LOC100910427	Protein LOC100910427	131
D3ZB30	Ptbp1	Polypyrimidine tract binding protein 1, isoform CRA_c	130
F1LM18	Ptbp1	Polypyrimidine tract-binding protein 1	130
Q00438	Ptbp1	Polypyrimidine tract-binding protein 1	130
P62828	Ran	GTP-binding nuclear protein Ran	130
Q921A3	Ubd	Ubiquitin D	129
G3V8D5	Pgls	6-phosphogluconolactonase	128
P85971	Pgls	6-phosphogluconolactonase	128
P25113	Pgam1	Phosphoglycerate mutase 1	128
F1M0E9	Non-identified	Uncharacterized protein	127
Q8CG45	Akr7a2	Aflatoxin B1 aldehyde reductase member 2	127
P18421	Psmb1	Proteasome subunit beta type-1	127
Q6PDW4	Psmb1	Proteasome subunit beta type	127
D4A7Q5	Ddx28	DEAD (Asp-Glu-Ala-Asp) box polypeptide 28	126
Q5XI38	Lcp1	Lymphocyte cytosolic protein 1	126
Q5XIE2	Mterf2	Transcription termination factor 2, mitochondrial	124
D3ZMQ7	Wdr74	Protein Wdr74	124
Q5XI36	Adgre5	CD97 molecule	123
Q5XID7	Armcx3	Armadillo repeat-containing X-linked protein 3	122
P69682	Necap1	Adaptin ear-binding coat-associated protein 1	122
D3ZM36	Il10rb	Protein Il10rb	120
Q6MG61	Clic1	Chloride intracellular channel protein 1	120

B1WC69	Nsun6	NOL1/NOP2/Sun domain family, member 6	119
Q5FWT9	Sike1	Suppressor of IKBKE 1	119
D3ZIZ6	Abcc12	Multidrug resistance-associated protein 9	118
F1M7G9	Abcc12	Multidrug resistance-associated protein 9	118
Q6Y306	Abcc12	Multidrug resistance-associated protein 9	118
O35460	Angpt1	Angiopoietin-1	118
D4A4D4	LOC290415	Uncharacterized protein	118
Q6AXV0	Pnkp	Polynucleotide kinase 3'-phosphatase	116
D3ZD15	Non-identified	Uncharacterized protein	116
P20070	Cyb5r3	NADH-cytochrome b5 reductase 3	115
Q6AYS8	Hsd17b11	Estradiol 17-beta-dehydrogenase 11	115
F1LUT3	LOC100912524	Protein LOC100912524	113
D3ZLA3	Cpne3	Copine 3 protein	112
O35077	Gpd1	Glycerol-3-phosphate dehydrogenase [NAD(+)], cytoplasmic	112
O89049	Txnrd1	Thioredoxin reductase 1, cytoplasmic	112
R9PXU4	Txnrd1	Thioredoxin reductase 1, cytoplasmic	112
D3ZR52	Lpcat4	Protein Lpcat4	112
G3V7G0	Dync1li1	Cytoplasmic dynein 1 light intermediate chain 1	112
Q9QXU8	Dync1li1	Cytoplasmic dynein 1 light intermediate chain 1	112
F1M5V8	Pilra	Protein LOC100910669	111
Q9ERE6	Mprip	Myosin phosphatase Rho-interacting protein	111
P55281	Cdh17	Cadherin-17	110
P97527	Cntn5	Contactin-5	108
F1M0F1	Onecut3	One cut domain family member	107
D3ZIK0	Glce	Protein Glce	107
D4A8C1	Kiz	Protein Kiz	107
Q5HZE4	Mri1	Methylthioribose-1-phosphate isomerase	107
D3ZRB1	Non-identified	Uncharacterized protein	107
M0RCA7	LOC687399	Protein LOC687399	107
Q5U2R4	Trmt10c	Mitochondrial ribonuclease P protein 1	106
D3ZKE6	Slmap	Sarcolemma associated protein	105
F1LPB3	Acsl5	Long-chain-fatty-acid--CoA ligase 5	105
O88813	Acsl5	Long-chain-fatty-acid--CoA ligase 5	105
G3V8Q2	Ina	Alpha-internexin	105
P23565	Ina	Alpha-internexin	105

Q3T1H2	Ngrn	Neugrin	104
Q7TP13	Gpt2	Cc2-5	104
Q811U3	Erc1	ELKS/Rab6-interacting/CAST family member 1	104
F1M173	Cntn5	Contactin-5	102
B1WC60	Pwwp2b	PWWP domain containing 2	102
F1LSL1	Purb	Transcription factor Pur-beta	101
D3ZY42	Tchp	Protein Tchp	101
G3V6P2	Dlst	Dihydrolipoamide S-succinyltransferase (E2 component of 2-oxo-glutarate complex), isoform CRA_a	100
Q01205	Dlst	Dihydrolipoyllysine-residue succinyltransferase component of 2-oxoglutarate dehydrogenase complex, mitochondrial	100
B5DEY0	Pls1	Pls1 protein	98
O70199	Ugdh	UDP-glucose 6-dehydrogenase	98
P97577	Fez1	Fasciculation and elongation protein zeta-1	96
D4A8D5	Flnb	Filamin, beta	65

The proteins identified are organized in a decrescent order of the score. The identification was performed according to UNIPROT database (http://www.uniprot.org/).

Supplementary Table S4. Proteins identified exclusively in the rats duodenum of the 10 ppm F group after chronic F exposure (ingestion of deionized water for 30 days).

Access Number	Gene name	Protein name description	Score
P13471	Rps14	40S ribosomal protein S14	1114
Q6PDV6	Rps14	Protein LOC100911847	1114
P48500	Tpi1	Triosephosphate isomerase	1041
P62859	Rps28	40S ribosomal protein S28	1016
Q5M9I5	Uqcrh	Cytochrome b-c1 complex subunit 6, mitochondrial	968
D4A0T0	Ndufb10	Protein Ndufb10	925
P62083	Rps7	40S ribosomal protein S7	761
M0R9D9	Non-identified	Uncharacterized protein	663

M0RD75	Rps6	Uncharacterized protein	629
P62755	Rps6	40S ribosomal protein S6	629
P36201	Crip2	Cysteine-rich protein 2	520
Q9Z144	Lgals2	Galectin-2	518
Q3KRD8	Eif6	Eukaryotic translation initiation factor 6	509
P00481	Otc	Ornithine carbamoyltransferase, mitochondrial	501
M0R5M7	RGD1563812	Protein RGD1560568	500
M0RB86	LOC683456	Protein LOC683456	500
Q5U3Y8	Btf3	Basic transcription factor 3	500
A7VJC2	Hnrnpa2b1	Heterogeneous nuclear ribonucleoproteins A2/B1	484
F1LM82	Hnrnpa2b1	Heterogeneous nuclear ribonucleoproteins A2/B1	484
F1LNF1	Hnrnpa2b1	Heterogeneous nuclear ribonucleoproteins A2/B1	484
M0R6J9	Hnrnpa2b1	Heterogeneous nuclear ribonucleoproteins A2/B1	484
D3ZGN8	LOC103690015	Uncharacterized protein	428
D3ZSH6	LOC100360843	Protein LOC100360843	428
D4A6G6	LOC100362339	Protein LOC100362339	428
F1LYF3	Rps19l1	Protein Rps19l1	428
F1M764	Non-identified	Uncharacterized protein	428
P17074	Rps19	40S ribosomal protein S19	428
Q9Z1Z9	Pdlim7	PDZ and LIM domain protein 7	426
D3ZWT8	Atp5h	ATP synthase subunit d, mitochondrial	399
P31399	Atp5h	ATP synthase subunit d, mitochondrial	399
D4A2K1	Hoga1	Protein Hoga1	383
D3ZXI2	Gm5611	Protein Gm5611	354
Q5XHZ0	Trap1	Heat shock protein 75 kDa, mitochondrial	340
D4A1Z2	Rpl26-ps2	Uncharacterized protein	330
M0R964	Non-identified	Uncharacterized protein	320
F1LNA9	Clps	Colipase	312
P17084	Clps	Colipase	312
P36972	Aprt	Adenine phosphoribosyltransferase	312
P21571	Atp5j	ATP synthase-coupling factor 6, mitochondrial	307
D3ZA50	Klhl15	Protein Klhl15	293
M0R4A9	RGD1561730	Protein RGD1561730	291

A0A096MJE5	Klhl15	Protein Klhl15	291
G3V983	Gstm1	Glutathione S-transferase Mu 1	278
P04905	Gstm1	Glutathione S-transferase Mu 1	278
F1M2R8	RGD1560936	60S ribosomal protein L13	273
P14604	Echs1	Enoyl-CoA hydratase, mitochondrial	268
G3V7B5	Prpsap1	Phosphoribosyl pyrophosphate synthase-associated protein 1	266
Q63468	Prpsap1	Phosphoribosyl pyrophosphate synthase-associated protein 1	266
F1M023	LOC685411	Uncharacterized protein	262
P62909	Rps3	40S ribosomal protein S3	257
D4A5L9	LOC679794	Protein LOC690675	255
P62898	Cycs	Cytochrome c, somatic	255
Q561R0	Mzb1	Marginal zone B- and B1-cell-specific protein	233
B0K008	Eif1	Eukaryotic translation initiation factor 1	221
B5DFN1	Eif1b	Eukaryotic translation initiation factor 1B	221
F1LWP8	LOC102551744	Protein LOC102551744	221
Q5BK48	Ttc5	Tetratricopeptide repeat protein 5	220
P52847	Sult1b1	Sulfotransferase family cytosolic 1B member 1	218
M0R3W8	Non-identified	Uncharacterized protein	208
M0R903	Non-identified	Uncharacterized protein	208
D3ZQV0	LOC100365995	Protein LOC100365995	207
F1LSJ2	Plin3	Uncharacterized protein	205
A0A096MJA0	Tcp1	RCG44919, isoform CRA_b	204
P28480	Tcp1	T-complex protein 1 subunit alpha	204
Q32Q55	Ces2h	Protein Ces2h	202
Q6MGA6	Psmb9	Proteasome subunit beta type	202
P07895	Sod2	Superoxide dismutase [Mn], mitochondrial	201
M0RAE7	Non-identified	Uncharacterized protein	197
D3ZCV5	Aldh1a7	Aldehyde dehydrogenase, cytosolic 1	197
P13601	Aldh1a7	Aldehyde dehydrogenase, cytosolic 1	197
P51647	Aldh1a1	Retinal dehydrogenase 1	194
D3ZVH2	RGD1560831	Protein RGD1560831	193
P63004	Pafah1b1	Platelet-activating factor acetylhydrolase IB subunit alpha	191
D3ZIF1	Agr3	Protein Agr3	190
F1LX46	Fmr1nb	Protein Fmr1nb	189
D4ABV5	Calm2	Calmodulin	188

A0A096MJW9	Ddx17	Protein Ddx17	187
I6L9G6	Tardbp	Protein Tardbp	186
M0R9K1	LOC100359916	Protein Gm7964	185
M0R5T1	Non-identified	Pyruvate kinase	183
O35531	Cd86	Cd86 antigen, isoform CRA_c	183
D3ZFR7	Pdlim3	PDZ and LIM domain 3, isoform CRA_b	179
Q66HS7	Pdlim3	PDZ and LIM domain protein 3	179
Q9JJ19	Slc9a3r1	Na(+)/H(+) exchange regulatory cofactor NHE-RF1	179
F1M0K0	Cadps2	Protein Cadps2	178
F1LYK8	Cadps2	Protein Cadps2	176
F1M068	Cadps2	Protein Cadps2	176
M0R705	Cadps2	Protein Cadps2	176
D4A1W5	Rbm4	Protein LOC100909948	176
P81155	Vdac2	Voltage-dependent anion-selective channel protein 2	172
F1LUV8	Non-identified	Uncharacterized protein	168
D1MCF1	Atp7a	Copper-transporting ATPase 1	165
F1M6D1	Non-identified	Uncharacterized protein	163
G3V6R7	Oxsm	3-oxoacyl-[acyl-carrier-protein] synthase, mitochondrial	162
Q9JLJ3	Aldh9a1	4-trimethylaminobutyraldehyde dehydrogenase	160
B1WBZ1	Efs	Embryonal Fyn-associated substrate	159
D3ZEN2	Non-identified	Glyceraldehyde-3-phosphate dehydrogenase	159
G3V6C4	Ugdh	UDP-glucose 6-dehydrogenase	158
F7EPE0	Psap	Sulfated glycoprotein 1	157
P10960	Psap	Sulfated glycoprotein 1	157
Q794E4	Hnrnpf	Heterogeneous nuclear ribonucleoprotein F	155
G3V9Z4	Aspdh	Putative L-aspartate dehydrogenase	154
Q5I0J9	Aspdh	Putative L-aspartate dehydrogenase	154
Q9Z214	Homer1	Homer protein homolog 1	153
Q5U2U8	Bag3	Bcl2-associated athanogene 3	152
D3ZM26	Mecom	Ecotropic viral integration site 1 (Predicted), isoform CRA_a	152
P45479	Ppt1	Palmitoyl-protein thioesterase 1	152
F1M5U6	Cadps2	Protein Cadps2	151
B5DFC3	Sec23a	Protein Sec23a	150
M0RAG7	Cadps2	Protein Cadps2	150
B0BN17	Cml5	Cml5 protein	150

Q66HF1	Ndufs1	NADH-ubiquinone oxidoreductase 75 kDa subunit, mitochondrial	148
Q64633	Ugt1a7c	UDP-glucuronosyltransferase 1-7	148
B2RYU7	Cbx5	Cbx5 protein	148
A0A096MJM1	Rhog	Protein Rhog	147
A0A096MK75	Rhog	Protein Rhog	147
Q32PX6	Rhog	Protein Rhog	147
D4A9L9	Erp27	Protein Erp27	147
M0R4U4	Non-identified	Triosephosphate isomerase	147
M0R613	Cd70	Protein Cd70	146
P46413	Gss	Glutathione synthetase	144
Q5U300	Uba1	Ubiquitin-like modifier-activating enzyme 1	141
P26376	ifitm3	Interferon-induced transmembrane protein 3	140
M0RA08	Plin3	Perilipin	138
A0A096MJ07	Krt9	Keratin, type I cytoskeletal 9	138
F1M7K4	Krt9	Keratin, type I cytoskeletal 9	138
Q8CIS9	Krt9	Keratin, type I cytoskeletal 9	138
G3V6U7	Msx1	Protein Msx1	136
D3ZJ50	Pkp3	Plakophilin 3 (Predicted), isoform CRA_a	135
Q6AYD5	Gspt1	G1 to S phase transition 1	135
P07323	Eno2	Gamma-enolase	135
Q64550	Ugt1a1	UDP-glucuronosyltransferase 1-1	131
Q9Z252	Lin7b	Protein lin-7 homolog B	130
M0R509	RGD1561998	Protein RGD1561998	129
Q4KM31	Limd2	LIM domain-containing protein 2	129
D3ZKW5	Pdik1l	PDLIM1 interacting kinase 1 like	129
F1LRA0	Pcdhga4	Protein LOC102557447	128
D3ZVD7	Kera	Keratocan	125
G3V618	Mapk13	Mitogen activated protein kinase 13	125
Q9WTY9	Mapk13	Mitogen-activated protein kinase 13	125
F1LVA9	Dock5	Protein Dock5	124
F1MA88	Dock5	Protein Dock5	124
Q2YDU3	Otud5	OTU domain-containing protein 5	124
Q4FZT0	Stoml2	Stomatin-like protein 2, mitochondrial	123
P13676	Apeh	Acylamino-acid-releasing enzyme	123
D4A4P4	Flad1	Protein Flad1	121

Q9EPJ1	Twist1	Protein Twist1	119
G3V9U1	Agap1	Centaurin, gamma 2	117
D4A318	Pld1	Phospholipase D1	114
F1LMG4	Pld1	Phospholipase D1	114
P70496	Pld1	Phospholipase D1	114
Q9WU82	Ctnnb1	Catenin beta-1	114
F1LWT0	Simc1	Protein Simc1	108
D3ZRM9	LOC100360491	60S ribosomal protein L13	107
P41123	Rpl13	60S ribosomal protein L13	107
B2RYN2	Fbxo31	F-box only protein 31	95
P0C5E3	Palld	Palladin	93
G3V9G5	Synm	Protein Synm	87
M0R6B1	Caprin2	Protein Caprin2	61
F1LWH5	Catsperg1	Protein Catsperg1	50
F1LNK0	Map2	Microtubule-associated protein	44
F1MAQ5	Map2	Microtubule-associated protein	44

The proteins identified are organized in a decrescent order of the score. The identification was performed according to UNIPROT database (http://www.uniprot.org/).

Supplementary Table S5. Proteins identified exclusively in the rats duodenum of the 50 ppm F group after chronic F exposure (ingestion of deionized water for 30 days).

**Nº de acesso da proteína**	**Gene**	**Nome da proteína**	**Score**
P13471	Rps14	40S ribosomal protein S14	1114
Q6PDV6	Rps14	Protein LOC100911847	1114
P48500	Tpi1	Triosephosphate isomerase	1041
D4A0T0	Ndufb10	Protein Ndufb10	925
M0RD75	Rps6	Uncharacterized protein	629
P62755	Rps6	40S ribosomal protein S6	629
Q07984	Ssr4	Translocon-associated protein subunit delta	535
Q3KRD8	Eif6	Eukaryotic translation initiation factor 6	509
M0RCH2	Glud1	Glutamate dehydrogenase 1, mitochondrial	498

A7VJC2	Hnrnpa2b1	Heterogeneous nuclear ribonucleoproteins A2/B1	484
F1LM82	Hnrnpa2b1	Heterogeneous nuclear ribonucleoproteins A2/B1	484
F1LNF1	Hnrnpa2b1	Heterogeneous nuclear ribonucleoproteins A2/B1	484
M0R6J9	Hnrnpa2b1	Heterogeneous nuclear ribonucleoproteins A2/B1	484
D3ZGN8	LOC103690015	Uncharacterized protein	428
D4A1G4	Cyb5a	Cytochrome b5	403
P00173	Cyb5a	Cytochrome b5	403
D4A2K1	Hoga1	Protein Hoga1	383
D3ZXI2	Gm5611	Protein Gm5611	354
M0R8U7	Spata17	Protein Spata17	349
P35704	Prdx2	Peroxiredoxin-2	337
F1LNA9	Clps	Colipase	312
P17084	Clps	Colipase	312
M0R8K1	Rnf187	E3 ubiquitin-protein ligase RNF187	302
P14604	Echs1	Enoyl-CoA hydratase, mitochondrial	268
P62909	Rps3	40S ribosomal protein S3	257
F1LWH8	LOC100911795	Protein LOC100911795	255
Q561R0	Mzb1	Marginal zone B- and B1-cell-specific protein	233
F1LSW7	Rpl14	60S ribosomal protein L14	223
Q63507	Rpl14	60S ribosomal protein L14	223
Q6TUH8	LOC306079	LRRGT00066	223
D4A0D6	Bcl7b	Protein Bcl7b	221
B0BN51	Snrpb	Small nuclear ribonucleoprotein-associated protein	215
P17136	Snrpb	Small nuclear ribonucleoprotein-associated protein B	215
P63164	Snrpn	Small nuclear ribonucleoprotein-associated protein N	215
Q5XIU9	Pgrmc2	Membrane-associated progesterone receptor component 2	201
M0RAE7	Non-identified	Uncharacterized protein	197
D3ZVH2	RGD1560831	Protein RGD1560831	193
M0RAB2	Gm20721	Protein Gm20721	192
O88637	Pcyt2	Ethanolamine-phosphate cytidylyltransferase	188
O89035	Slc25a10	Mitochondrial dicarboxylate carrier	187
M0R9C1	Non-identified	Uncharacterized protein	187
Q5BJY3	Ino80c	INO80 complex subunit C	183
D3ZYU0	Non-identified	Enolase	180
D3ZFR7	Pdlim3	PDZ and LIM domain 3, isoform CRA_b	179

Q66HS7	Pdlim3	PDZ and LIM domain protein 3	179
M0RDH6	LOC100909427	Protein LOC100909427	171
P18757	Cth	Cystathionine gamma-lyase	166
Q9EQS4	Cth	Cystathionase (Cystathionine gamma-lyase)	166
P13437	Acaa2	3-ketoacyl-CoA thiolase, mitochondrial	164
M0R6R9	LOC102549011	Protein LOC102549011	162
D4A3P1	Ubqln4	Protein Ubqln4	160
D3ZRL6	Fcar	Protein Fcar	154
P15429	Eno3	Beta-enolase	153
D3ZZP2	Rab39a	Protein Rab39a	152
D3ZBG8	Papd5	PAP associated domain containing 5	152
G3V9U2	Acaa2	3-ketoacyl-CoA thiolase, mitochondrial	152
A0A096MJZ0	Dnal1	Protein Dnal1	150
F1LMV0	Dnalc1	Protein Dnal1	150
Q9WTT6	Gda	Guanine deaminase	149
P46413	Gss	Glutathione synthetase	144
Q9Z0V5	Prdx4	Peroxiredoxin-4	142
M0R7F0	Htra3	Serine protease HTRA3	139
Q9EPH8	Pabpc1	Polyadenylate-binding protein 1	138
F7F379	Armc1	Armadillo repeat containing 1	138
P25086	Il1rn	Interleukin-1 receptor antagonist protein	137
A0A096MK39	Hsf4	Protein Hsf4	136
D4A5A8	Hsf4	Heat shock transcription factor 4 (Predicted), isoform CRA_c	136
P07323	Eno2	Gamma-enolase	135
Q5XIR8	Clhc1	Clathrin heavy chain linker domain-containing protein 1	134
D3ZR12	Sntg2	Protein Sntg2	133
O08701	Arg2	Arginase-2, mitochondrial	132
D3ZD13	LOC100911261	Protein LOC100911261	131
Q5U204	Lamtor3	Ragulator complex protein LAMTOR3	131
Q64550	Ugt1a1	UDP-glucuronosyltransferase 1-1	131
D3Z9G0	Traf3	Protein Traf3	129
F1LXE7	Non-identified	Uncharacterized protein	128
F7ELD4	Ugt1a5	Protein LOC100912040	128
Q6T5E7	Ugt1a1	Protein LOC100912040	128
Q6T5E9	Ugt1a1	Protein LOC100912040	128

Q6T5F1	Ugt1a1	Protein LOC100912040	128
Q6T5F2	Ugt1a1	Protein LOC100912040	128
D3ZWW3	Rasal3	Protein Rasal3	127
D3ZLV3	Ybx2	Protein Ybx2	127
D3ZKY9	Spata22	Protein Spata22	125
G3V8F4	Scrn3	Protein Scrn3	125
D3ZTQ3	Non-identified	Uncharacterized protein	125
F1M4R7	Cspg5	Chondroitin sulfate proteoglycan 5	121
Q9ERQ6	Cspg5	Chondroitin sulfate proteoglycan 5	121
Q3B7U2	Taf1a	TATA box-binding protein-associated factor RNA polymerase I subunit A	120
Q9JKB7	Gda	Guanine deaminase	120
Q5MPP5	Ly49i7	Immunoreceptor Ly49i7	120
P05426	Rpl7	60S ribosomal protein L7	120
D3ZRF7	Hsh2d	Protein Hsh2d	119
A0A096MJV3	Hsf4	Protein Hsf4	119
D3ZCQ0	Col19a1	Protein Col19a1	119
F1LQX9	Nploc4	Nuclear protein localization protein 4 homolog	118
Q9ES54	Nploc4	Nuclear protein localization protein 4 homolog	118
Q4PJT6	Spata24	Spermatogenesis-associated protein 24	118
F1LRJ8	Lmx1a	Protein Lmx1a	117
D3ZZK1	LOC100359563	Protein LOC100359563	116
M0RAK8	LOC100912386	Protein LOC100912386	116
P60868	Rps20	40S ribosomal protein S20	116
D3ZTX5	Efhb	EF hand domain family, member B (Predicted), isoform CRA_b	115
M0RAW8	Non-identified	Uncharacterized protein	115
B0K031	Rpl7	60S ribosomal protein L7	115
F1M087	Non-identified	Uncharacterized protein	115
P02454	Col1a1	Collagen alpha-1(I) chain	114
Q5M9G8	Dcaf11	DDB1- and CUL4-associated factor 11	113
D3ZRD2	Ccdc15	Protein Ccdc15	112
P13803	Etfa	Electron transfer flavoprotein subunit alpha, mitochondrial	112
F1LM66	Eftud2	Protein Eftud2	111
B2GV10	Efhb	Efhb protein	111
F1LPS4	Cyp11b2	Cytochrome P450 11B2, mitochondrial	110
P30099	Cyp11b2	Cytochrome P450 11B2, mitochondrial	110

P30100	Cyp11b3	Cytochrome P450 11B3, mitochondrial	110
D3ZGK3	Tbata	Protein Tbata	110
F1LZT3	Tbata	Protein Tbata	110
Q6Q0N1	Cndp2	Cytosolic non-specific dipeptidase	110
D3ZFK6	Atg16l1	Protein Atg16l1	110
G3V7H6	Arg2	Arginase	109
D4AAD0	Nxpe5	Protein Nxpe5	108
F1M0C7	Nxpe5	Protein Nxpe5	108
Q5U2Y2	Satb1	DNA-binding protein SATB	107
Q4V7E3	Zbed3	Protein Zbed3	106
Q66X93	Snd1	Staphylococcal nuclease domain-containing protein 1	106
Q6AXT5	Rab21	Ras-related protein Rab-21	106
Q9JHZ4	Gripap1	GRIP1-associated protein 1	105
B5DF80	Pabpc6	Poly(A) binding protein, cytoplasmic 3	105
Q4V8G8	Tekt3	Tektin-3	104
D3ZN01	RGD1307830	Protein RGD1307830	103
D3ZV63	RGD1307830	Protein RGD1307830	103
F1LQS3	Rpl6	60S ribosomal protein L6	103
H7C5Y5	Rpl6	60S ribosomal protein L6	103
P21533	Rpl6	60S ribosomal protein L6	103
Q5U2S7	Psmd3	Proteasome (Prosome, macropain) 26S subunit, non-ATPase, 3	102
Q9R1J4	Myoc	Myocilin	100
D4A455	Pcdhga10	Protein Pcdhga10	99
D4A4D7	E2f7	Transcription factor E2F7	90
M0R5L4	Non-identified	Uncharacterized protein	83
A0A096UWG9	Dgke	Protein Dgke	80
M0R7G2	Non-identified	Uncharacterized protein	77

The proteins identified are organized in a decrescent order of the score. The identification was performed according to UNIPROT database (http://www.uniprot.org/).

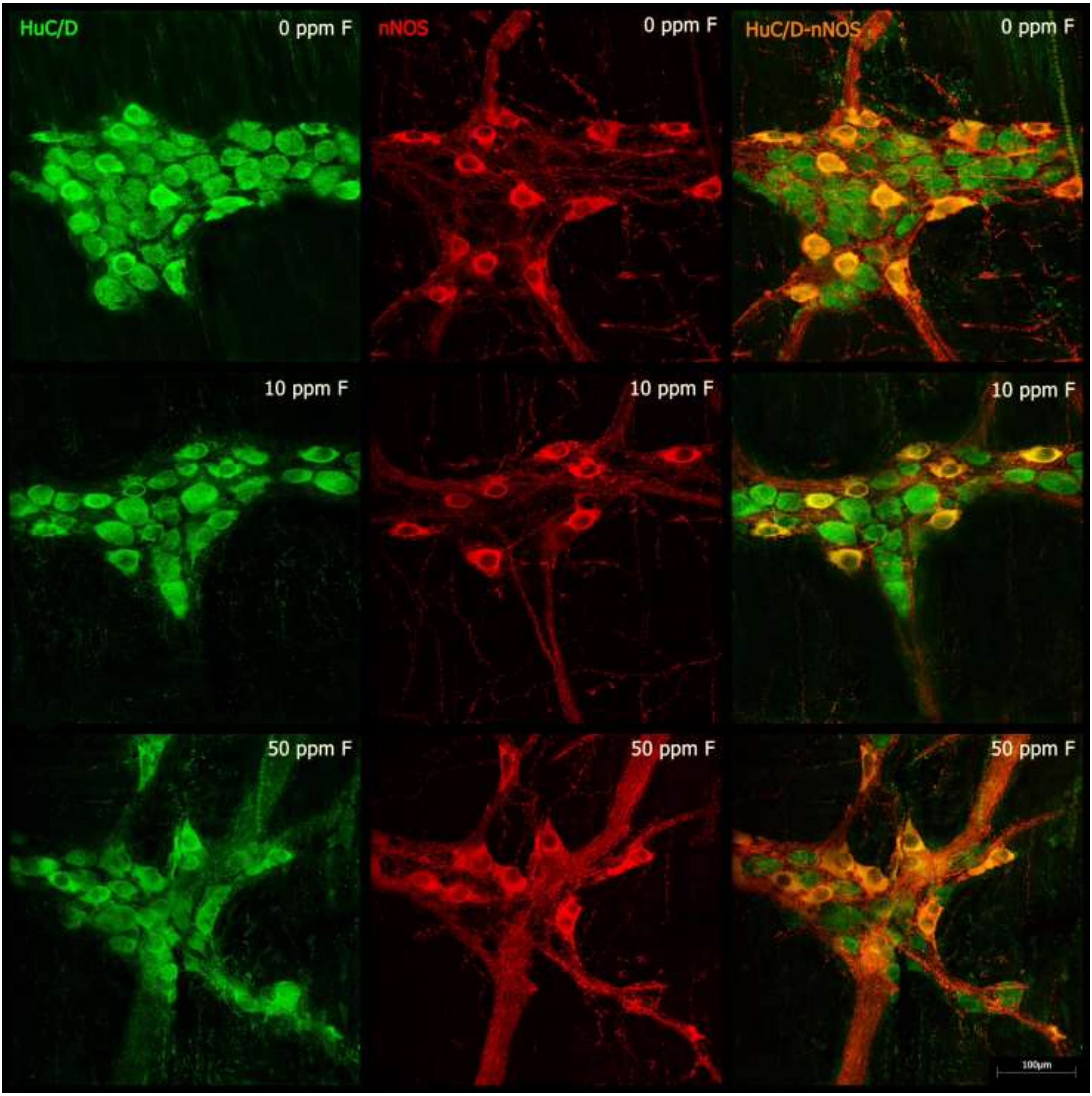

Supplementary Figure 1. Photomicrography of myenteric neurons of the rats duodenum after chronic F exposure stained for HuC/D (green), nNOS (red), and double-labeling (HuC/D and nNOS). Control Group: HuC/D-IR neurons (A), nNOS-IR neurons (B), and double labeling (HuC/D and nNOS neurons) (C). 10 ppm F Group: HuC/D-IR neurons(C), nNOS-IR neurons (D), and double labeling (HuC/D and nNOS neurons) (E). 50 ppm F Group: HuC/D-IR neurons (F), nNOS-IR neurons (G), and double labeling (HuC/D and nNOS neurons) (H). 20X Objective.

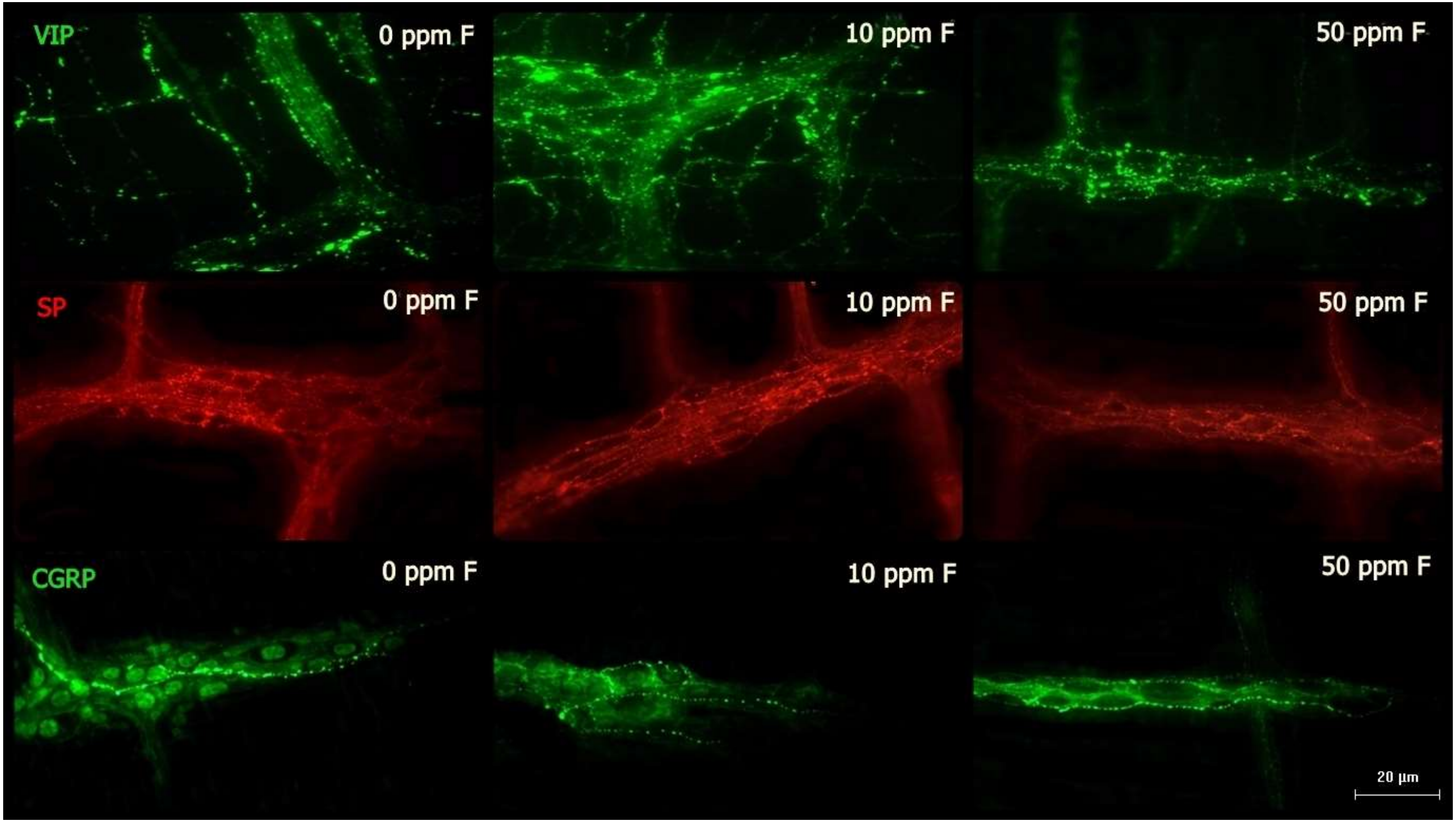

Supplementary Figure 2. Photomicrography of myenteric varicosities of the rats duodenum after chronic F exposure. VIP-IR varicosities: Control Group (A), 10 ppm F Group (B), 50 ppm F Group (C). CGRP-IR varicosities: Control Group (D), 10 ppm F Group (E), 50 ppm F Group (F). SP-IR varicosities: Control Group (G), 10 ppm F Group (H), 50 ppm F Group (I). 40X Objective.
